# Comparative real-world pharmacovigilance analysis of adverse event signals associated with six approved CAR-T products using the FAERS database

**DOI:** 10.3389/fphar.2026.1913235

**Published:** 2026-10-07

**Authors:** Shengyuan Xiao, Ying Li, Hui Wang, Longcai Tong, Rong Li

**Affiliations:** 1 Hunan Provincial Key Laboratory of Basic and Clinical Pharmacological Research of Gastrointestinal Cancer, Department of Pharmacy, Institute of Pharmacy and Pharmacology, The Second Affiliated Hospital, University of South China, Hengyang, Hunan, China; 2 Hengyang Medical School, University of South China, Hengyang, Hunan, China

**Keywords:** adverse events, CAR-T, FAERS, pharmacovigilance, real-world study

## Abstract

**Objective:**

Although several CAR-T products have been approved for hematologic malignancies, their post-marketing adverse-event reporting patterns may not be interchangeable. This study compared FAERS-derived positive signals across approved CAR-T products to distinguish recurrent class-related toxicities from product-skewed reporting patterns.

**Methods:**

FAERS reports from Q1 2014 to Q2 2025 were reviewed for CAR-T products. Demographic characteristics were summarized for seven products, whereas PT- and SOC-level signal analyses were restricted to six products with sufficient signal data: Breyanzi, Kymriah, Tecartus, Yescarta, Abecma, and Carvykti. Positive signals were detected using ROR, PRR, and BCPNN algorithms. After predefined exclusion of nonclinical or non-AE terms, indication- or disease-progression-related terms, procedure- or investigation-related terms, efficacy- or persistence-related terms, and product-quality-related terms, eligible signals were compared at the PT and SOC levels. Nonspecific MedDRA-coded PTs were retained if they met the predefined signal criteria, but they were interpreted descriptively and were not used to define product-level profile labels.

**Results:**

CRS was consistently among the leading PT-level signals across all six products. Yescarta and Tecartus showed higher reporting of ICANS and neurotoxicity-related PTs. Kymriah was more strongly represented by infection-, immune-, and hematologic-related signals, whereas Carvykti showed several neurologic PTs including Parkinsonism, cranial nerve paralysis, facial paralysis, and Bell’s palsy. Abecma showed relatively high-ranking systemic symptom-related PTs, including fatigue, headache, and decreased appetite. At the SOC level, nervous system disorders and infections and infestations were among the most frequently represented categories, but their proportions differed across products.

**Conclusion:**

FAERS-based signal detection showed a common CAR-T reporting backbone centered on CRS, ICANS/neurotoxicity, hypotension, pyrexia, infection-related events, and hematologic events, together with product-skewed signal patterns. These findings represent disproportionality signals rather than comparative safety rankings, and FAERS data cannot establish causal relationships between CAR-T products and adverse events.

## 1 Introduction

Chimeric antigen receptor T-cell (CAR-T) therapy has become an important therapeutic strategy for patients with relapsed or refractory hematologic malignancies and has achieved durable remission in a subset of patients, as demonstrated in pivotal studies of several approved CAR-T products (Abramson et al., 2020; Schuster et al., 2019; Wang et al., 2020; Neelapu et al., 2017; Munshi et al., 2021; Berdeja et al., 2021). However, treatment-related toxicities remain a major challenge limiting its broader clinical application (Morris et al., 2022; Schubert et al., 2021). In addition to typical class-effect toxicities such as cytokine release syndrome (CRS) and immune effector cell-associated neurotoxicity syndrome (ICANS), differences among products may also exist in terms of infection, myelosuppression, and neurologic injury (Morris et al., 2022; Schubert et al., 2021; Lee et al., 2019; Guo et al., 2024).

Currently approved CAR-T products differ in target design, co-stimulatory domains, cellular expansion kinetics, and *in vivo* persistence, which may contribute to heterogeneity in their toxicity profiles (Abramson et al., 2020; Schuster et al., 2019; Wang et al., 2020; Neelapu et al., 2017; Munshi et al., 2021; Berdeja et al., 2021; Morris et al., 2022; Schubert et al., 2021). Previous studies have mainly focused on individual products or specific categories of toxicities, whereas systematic comparisons of the real-world safety spectra across different CAR-T products remain relatively limited (Guo et al., 2024). The FDA Adverse Event Reporting System (FAERS) database provides large-scale real-world data for post-marketing drug safety evaluation and is particularly suitable for generating hypotheses regarding rare or product-specific safety signals (Cutroneo et al., 2024). Previous FAERS-based pharmacovigilance research has shown that CAR-T-cell therapy is associated with a broad spectrum of reported toxicities across multiple organ systems, including CRS, ICANS, infection-related events, hematologic toxicities, and neurologic toxicities (Guo et al., 2024; Liu et al., 2026).

To address this gap, we conducted a comparative FAERS-based pharmacovigilance analysis of six CAR-T products using an integrated framework combining PT-level signal ranking, SOC-level signal distribution, and cross-product overlap patterns. Unlike previous studies focusing primarily on individual products or selected toxicity categories, the present analysis was designed to characterize both recurrent class-related signals and product-skewed reporting patterns across multiple CAR-T therapies. To improve clinical interpretability, predefined PT-exclusion rules were applied to remove non-AE terms and terms related to underlying indications or disease progression, lack of efficacy or treatment response, procedures or investigations, pharmacodynamic persistence, and product-quality issues before product-level comparisons were interpreted.

## 2 Materials and methods

### 2.1 Data source

This study used AE reports extracted from FAERS for CAR-T products. FAERS is a spontaneous reporting database and was used here for signal detection and descriptive comparison only. Demographic analyses included seven products: Aucatzyl, Breyanzi, Kymriah, Tecartus, Yescarta, Abecma, and Carvykti. Preferred term (PT)- and system organ class (SOC)-level analyses were restricted to six products with sufficient positive-signal data: Breyanzi, Kymriah, Tecartus, Yescarta, Abecma, and Carvykti. The overall workflow and analytic cohorts are shown in Figure 1.

**FIGURE 1:**
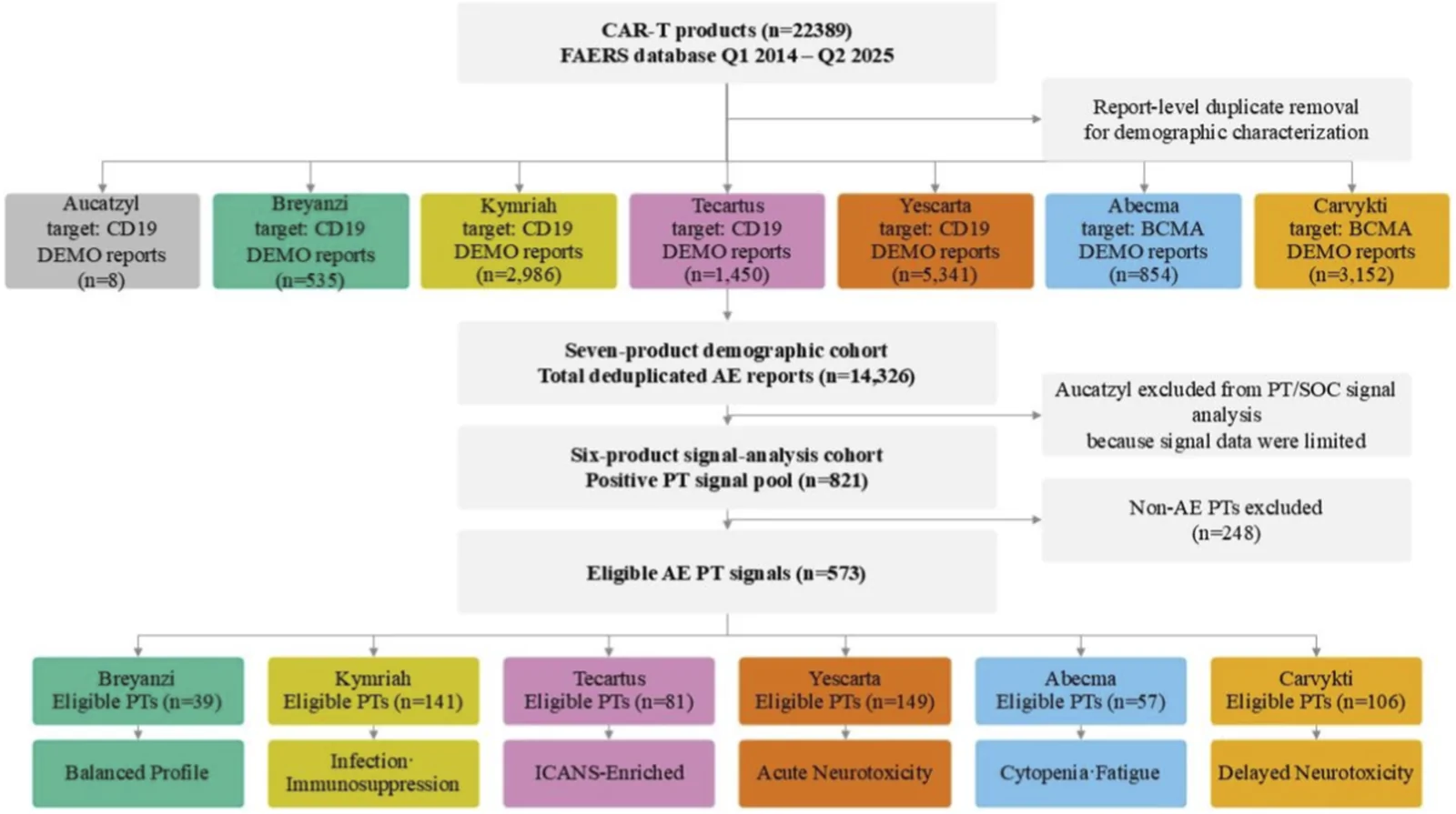
Study flow and analytic overview of CAR-T product cohorts and signal profiles. The bottom row shows descriptive product-level profile labels derived from clinically interpretable eligible PT-level signals, SOC-level distributions, forest plots, and cross-product overlap patterns. These labels should be interpreted as shorthand descriptions of reporting-signal patterns rather than as incidence-based or causality-based toxicity phenotypes.

### 2.2 Signal detection and data processing

FAERS reports from the first quarter of 2014 through the second quarter of 2025 were included in the analysis. Duplicate and follow-up reports were removed before signal detection. For records sharing the same CASEID, the report with the most recent FDA_DT was retained; when both CASEID and FDA_DT were identical, the record with the largest PRIMARYID was retained. Only reports in which the CAR-T product was designated as the primary suspect (PS) were included. Reports with missing demographic information were retained, and missing values were not imputed. Missing age or sex was categorized separately in the corresponding descriptive and subgroup analyses. Adverse events were standardized at the Preferred Term (PT) and System Organ Class (SOC) levels according to the Medical Dictionary for Regulatory Activities (MedDRA), version 25.0.

For each CAR-T product, PT-level disproportionality analyses were performed using three algorithms: the reporting odds ratio (ROR), proportional reporting ratio (PRR), and Bayesian confidence propagation neural network (BCPNN) (Cutroneo et al., 2024; Evans et al., 2001; Rothman et al., 2004; Bate et al., 1998). The fourfold contingency table was constructed at the drug–PT pair level. In the primary analysis, ROR was used as the principal criterion for signal detection, and a drug–PT pair was considered a positive signal when it met the predefined ROR criterion listed in Table 1. PRR and BCPNN were calculated as complementary disproportionality measures.

**TABLE 1:** Formulas and signal detection criteria for reporting odds ratio (ROR), proportional reporting ratio (PRR), and Bayesian confidence propagation neural network (BCPNN).

Algorithms	Equation	Criteria	​
ROR	ROR = ad/bc 95% CI = exp[ln(ROR) ± 1.96√(1/a +1/b + 1/c + 1/d)]	Lower limit of 95% CI > 1, a ≥3	​
PRR	PRR = a(c + d)/c(a + b) *χ* ^*2*^ = [(ad - bc)^2](a + b + c + d)/[(a + b) (c + d) (a + c) (b + d)]	PRR >2, *χ* ^*2*^ > 4, a ≥3	​
BCPNN	IC = log_2_ [a(a + b + c + d)/((a + b) (a + c))] 95% CI = E(IC) ± 2√V(IC)	IC025 > 0	​

Fourfold table of disproportionality measures	Target AE	Other AE	Total
Target drugs	a	b	a + b
Other drugs	c	d	c + d
Total	a + c	b + d	a + b + c + d

After initial signal detection, PTs were further screened to remove terms unlikely to represent interpretable adverse-event signals, including nonclinical or non-AE terms, laboratory investigation terms, surgical or medical procedure terms, underlying indications or disease progression, lack of efficacy or treatment response, CAR-T persistence or other pharmacodynamic durability indicators, and product quality issues. Incomplete case records that could not support PT-level interpretation were also excluded. PT screening was performed by three investigators, and borderline or uncertain PTs were discussed until consensus was reached before the final inclusion or exclusion decision. Nonspecific but formally coded MedDRA PTs were not removed solely because of limited clinical specificity if they met the primary ROR-based signal criterion; these terms were retained for descriptive reporting and tabulation but were not considered when characterizing product-level reporting patterns. Eligible PT signals were then ranked by reporting frequency, and the top 30 PTs for each product were used for the main comparative visualizations. For the subsequent subgroup analyses, a more stringent three-algorithm concordance criterion was applied, whereby a drug–PT pair was considered a positive signal only when the predefined thresholds for ROR, PRR, and BCPNN listed in Table 1 were simultaneously satisfied.

### 2.3 Statistical analysis and visualization

Descriptive analyses were performed for sex, age, reporting year, and geographic distribution of each product. The frequencies and RORs (95% confidence intervals [95% CI]) of the top 30 PT-level signals were summarized, and visualization analyses were conducted using bubble plots, forest plots, and a network diagram. In addition, eligible PT-level signal distribution patterns among different products were compared at the SOC level.

## 3 Results

### 3.1 Demographic characteristics of AE reports

The demographic characteristics of adverse event (AE) reports associated with seven CAR-T products in the FAERS database are presented in Figure 2 and Table 2. The United States was the primary source of AE reports for all products. Differences were observed among products with respect to sex, age, and reporting-year distributions. Among the included products, Yescarta accounted for the largest number of AE reports, followed by Carvykti, whereas Aucatzyl had the fewest reports.

**FIGURE 2:**
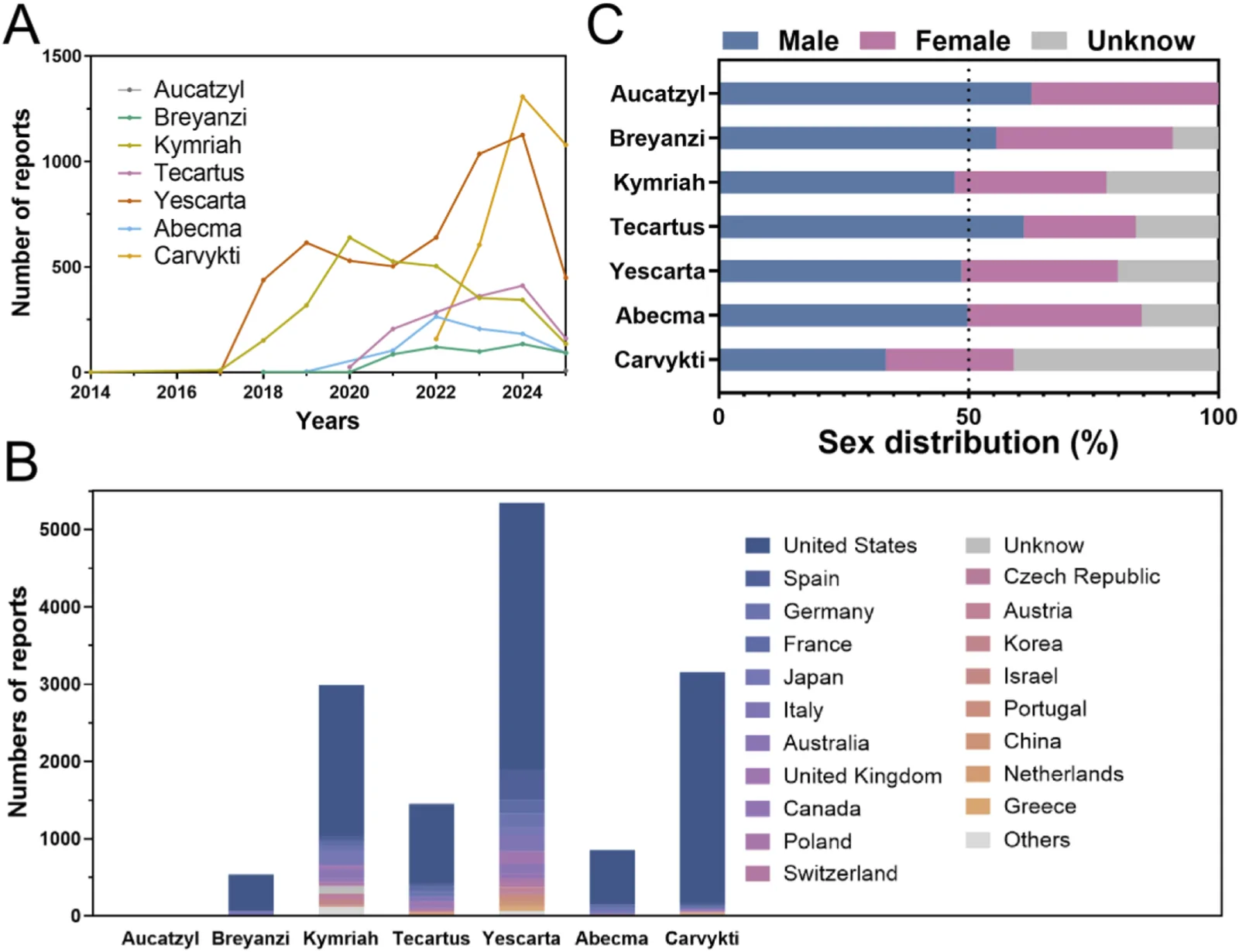
Demographic characteristics of adverse event reports in the FAERS database. **(A)** Annual number of reports for seven CAR-T products; **(B)** Geographic distribution of reports for the seven products; **(C)** Sex distribution of reports for the seven products.

**TABLE 2:** Demographic characteristics of AE reports associated with seven CAR-T products in the FAERS database.

Characteristic	Aucatzyl		Breyanzi		Kymriah		Tecartus		Yescarta		Abecma		Carvykti	
	Category	*n* (%)	Category	*n* (%)	Category	*n* (%)	Category	*n* (%)	Category	*n* (%)	Category	*n* (%)	Category	*n* (%)
Sex	Male	5 (62.5)	Male	297(55.5)	Male	1409 (47.2)	Male	885 (61.0)	Male	2592 (48.5)	Male	425 (49.8)	Male	1053 (33.4)
	Female	3 (37.5)	Female	189 (35.3)	Female	908 (30.4)	Female	325 (22.4)	Female	1674 (31.3)	Female	298 (34.9)	Female	808 (25.6)
	Unknown	0 (0)	Unknown	49 (9.2)	Unknown	669 (22.4)	Unknown	240 (16.6)	Unknown	1075 (20.1)	Unknown	131 (15.3)	Unknown	1291 (41.0)
Age (years)	<18	0(0)	<18	1(0.2)	<18	637 (21.3)	<18	8 (0.6)	<18	11 (0.2)	<18	0(0)	<18	0(0)
	18-<65	6(75.0)	18-<65	147(27.5)	18-<65	731 (24.5)	18-<65	485 (33.4)	18-<65	2134(40.0)	18-<65	247(28.9)	18-<65	350 (11.1)
	65–85	1(12.5)	65–85	316(59.1)	65–85	488 (16.3)	65–85	480(33.1)	65–85	1414(26.5)	65–85	388(45.4)	65–85	519 (16.5)
	>85	0(0)	>85	5(0.9)	>85	5 (0.2)	>85	2 (0.1)	>85	9 (0.2)	>85	5 (0.6)	>85	2 (0.1)
	Missing	1(12.5)	Missing	66(12.3)	Missing	1125(37.7)	Missing	475(32.8)	Missing	1773(33.2)	Missing	214(25.1)	Missing	2281(72.4)
Reporting year	2014	0(0)	2014	0(0)	2014	1 (0.0)	2014	0(0)	2014	0(0)	2014	0(0)	2014	0(0)
	2015	0(0)	2015	0(0)	2015	0(0)	2015	0(0)	2015	0(0)	2015	0(0)	2015	0(0)
	2016	0(0)	2016	0(0)	2016	0(0)	2016	0(0)	2016	0(0)	2016	0(0)	2016	0(0)
	2017	0(0)	2017	0(0)	2017	10 (0.3)	2017	0(0)	2017	1 (0.0)	2017	0(0)	2017	0(0)
	2018	0(0)	2018	1(0.2)	2018	152 (5.1)	2018	0(0)	2018	438 (8.2)	2018	0(0)	2018	0(0)
	2019	0(0)	2019	0(0)	2019	318 (10.6)	2019	0(0)	2019	616 (11.5)	2019	4 (0.5)	2019	0(0)
	2020	0(0)	2020	1(0.2)	2020	640 (21.4)	2020	25 (1.7)	2020	530 (9.9)	2020	0(0)	2020	0(0)
	2021	0(0)	2021	86(16.1)	2021	527 (17.6)	2021	206(14.2)	2021	504 (9.4)	2021	104(12.2)	2021	0(0)
	2022	0(0)	2022	120(22.4)	2022	505 (16.9)	2022	285(19.7)	2022	641 (12.0)	2022	264(30.9)	2022	159 (5.0)
	2023	0(0)	2023	99(18.5)	2023	353 (11.8)	2023	362(25.0)	2023	1036(19.4)	2023	207(24.2)	2023	606 (19.2)
	2024	0(0)	2024	135(25.2)	2024	344 (11.5)	2024	411(28.3)	2024	1126(21.1)	2024	183(21.4)	2024	1308(41.5)
	2025	8(100)	2025	93(17.4)	2025	136 (4.6)	2025	161(11.1)	2025	449 (8.4)	2025	92 (10.8)	2025	1079(34.2)
Geographical distribution	US	8(100)	US	462(86.4)	US	1952(65.4)	US	1034(71.3)	US	3440(64.4)	US	702(82.2)	US	2997(95.1)
	Spain	0(0)	Spain	1(0.2)	Spain	57(1.9)	Spain	34(2.3)	Spain	394(7.4)	Spain	2(0.2)	Spain	22(0.7)
	France	0(0)	France	4(0.7)	France	65(2.2)	France	69(4.8)	France	183(3.4)	France	51(6.0)	France	12(0.4)
	Germany	0(0)	Germany	3(0.6)	Germany	65(2.2)	Germany	59(4.1)	Germany	181(3.4)	Germany	19(2.2)	Germany	35(1.1)
	Japan	0(0)	Japan	49(9.2)	Japan	163(5.5)	Japan	0(0)	Japan	91(1.7)	Japan	52(6.1)	Japan	0(0)
	Italy	0(0)	Italy	2(0.4)	Italy	38(1.3)	Italy	65(4.5)	Italy	217(4.1)	Italy	1(0.1)	Italy	5(0.2)
​	UK	0(0)	UK	1(0.2)	UK	37(1.2)	UK	52(3.6)	UK	168(3.1)	UK	1(0.1)	UK	8(0.3)
​	Australia	0(0)	Australia	0(0)	Australia	127(4.3)	Australia	10(0.7)	Australia	118(2.2)	Australia	0(0)	Australia	10(0.3)
	Canada	0(0)	Canada	2(0.4)	Canada	46(1.5)	Canada	27(1.9)	Canada	62(1.2)	Canada	0(0)	Canada	1(0.0)
	Poland	0(0)	Poland	0(0)	Poland	42(1.4)	Poland	14(1.0)	Poland	71(1.3)	Poland	0(0)	Poland	4(0.1)
	Switzerland	0(0)	Switzerland	3(0.6)	Switzerland	12(0.4)	Switzerland	16(1.1)	Switzerland	45(0.8)	Switzerland	23(2.7)	Switzerland	1(0)
	Unknown	0(0)	Unknown	0(0)	Unknown	95(3.2)	Unknown	0(0)	Unknown	1(0.0)	Unknown	1(0.1)	Unknown	0(0)
	Austria	0(0)	Austria	4(0.7)	Austria	12(0.4)	Austria	14(1.0)	Austria	57(1.1)	Austria	1(0.1)	Austria	5(0.2)
	CZ	0(0)	CZ	0(0)	CZ	57(1.9)	CZ	4(0.3)	CZ	25(0.5)	CZ	0(0)	CZ	3(0.1)
	Korea	0(0)	Korea	0(0)	Korea	75(2.5)	Korea	0(0)	Korea	0(0)	Korea	0(0)	Korea	1(0)
	Israel	0(0)	Israel	0(0)	Israel	14(0.5)	Israel	5(0.3)	Israel	45(0.8)	Israel	0(0)	Israel	8(0.3)
	Portugal	0(0)	Portugal	0(0)	Portugal	0(0)	Portugal	7(0.5)	Portugal	60(1.1)	Portugal	0(0)	Portugal	0(0)
	China	0(0)	China	0(0)	China	0(0)	China	3(0.2)	China	50(0.9)	China	0(0)	China	12(0.4)
	Netherlands	0(0)	Netherlands	0(0)	Netherlands	9(0.3)	Netherlands	15(1.0)	Netherlands	24(0.4)	Netherlands	0(0)	Netherlands	7(0.2)
	Greece	0(0)	Greece	0(0)	Greece	1(0)	Greece	12(0.8)	Greece	36(0.7)	Greece	0(0)	Greece	0(0)
	Others	0(0)	Others	4(0.7)	Others	119(4.0)	Others	10(0.7)	Others	73(1.4)	Others	1(0.1)	Others	21(0.7)

US, UK, and CZ, denote the United States, the United Kingdom, and the Czech Republic, respectively.

The extent of missing demographic information varied substantially across products. Missing age information accounted for 12.5%, 12.3%, 37.7%, 32.8%, 33.2%, 25.1%, and 72.4% of reports for Aucatzyl, Breyanzi, Kymriah, Tecartus, Yescarta, Abecma, and Carvykti, respectively. Unknown sex accounted for 0%, 9.2%, 22.4%, 16.6%, 20.1%, 15.3%, and 41.0% of reports, respectively. Missing demographic values were not imputed and were retained as separate missing/unknown categories in the descriptive analyses.

#### 3.1.1 Demographic characteristics table

Table 2 provides detailed information on the sex, age, reporting year, and geographic distribution of AE reports associated with the seven CAR-T products.

### 3.2 Identification and analysis of positive AE signals at the PT level

After application of the predefined PT-exclusion rules, the numbers of eligible PT-level positive AE signals were 39 for Breyanzi, 141 for Kymriah, 81 for Tecartus, 149 for Yescarta, 57 for Abecma, and 106 for Carvykti. The complete eligible signal lists are provided in Supplementary Tables S1–S6, and the top 30 PT-level signals for each product are summarized in Figure 3 and Table 3.

**FIGURE 3:**
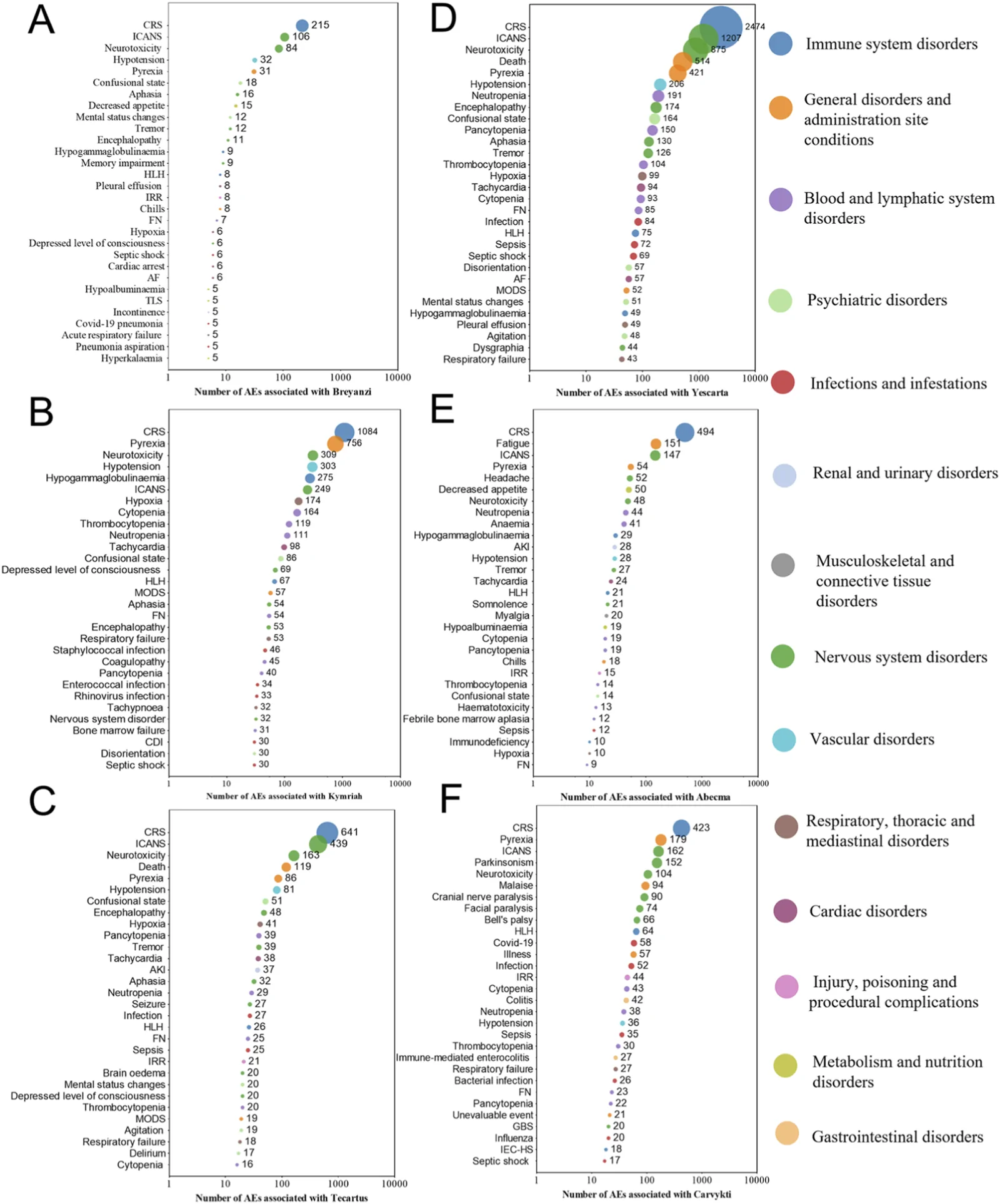
Top 30 PT-level positive AE signals and corresponding SOC classifications for six CAR-T products in the FAERS database. **(A)** Breyanzi; **(B)** Kymriah; **(C)** Tecartus; **(D)** Yescarta; **(E)** Abecma; and **(F)** Carvykti. CRS, cytokine release syndrome; ICANS, immune effector cell-associated neurotoxicity syndrome; AKI, acute kidney injury; HLH, haemophagocytic lymphohistiocytosis; IRR, infusion-related reaction; FN, febrile neutropenia; AF, atrial fibrillation; TLS, tumour lysis syndrome; MODS, multiple organ dysfunction syndrome; GBS, Guillain-Barré syndrome; CDI, Clostridium difficile infection; IEC-HS, immune effector cell-associated HLH-like syndrome.

**TABLE 3:** Top 30 positive AE signals associated with six CAR-T products in the FAERS database.

Breyanzi			Kymriah			Tecartus			Yescarta			Abecma			Carvykti		
PT	*n* (%)	ROR (95%CI)	PT	*n* (%)	ROR (95% CI)	PT	*n* (%)	ROR (95% CI)	PT	*n* (%)	ROR (95% CI)	PT	*n* (%)	ROR (95% CI)	PT	*n* (%)	ROR (95% CI)
Cytokine release syndrome	215 (15.60)	741.51 (640.44–858.53)	Cytokine release syndrome	1084 (7.52)	350.05 (328.2–373.36)	Cytokine release syndrome	641 (15.49)	362.82 (332.8–395.55)	Cytokine release syndrome	2474 (17.13)	1007.23 (960.68–1056.03)	Cytokine release syndrome	494 (20.20)	1037.73 (938.73–1147.17)	Cytokine release syndrome	423 (7.65)	337.63 (305.29–373.4)
Immune effector cell-associated neurotoxicity syndrome	106 (7.69)	1,142.69 (934.88–1396.69)	Pyrexia	756 (5.25)	9.89 (9.19–10.65)	Immune effector cell-associated neurotoxicity syndrome	439 (10.61)	743.51 (669.63–825.54)	Immune effector cell-associated neurotoxicity syndrome	1207 (8.36)	1772.44 (1652.26–1901.35)	Fatigue	151 (6.17)	5.23 (4.44–6.17)	Pyrexia	179 (3.24)	5.96 (5.14–6.92)
Neurotoxicity	84 (6.10)	245.12 (196.46–305.85)	Neurotoxicity	309 (2.14)	84.13 (75.07–94.29)	Neurotoxicity	163 (3.94)	124.96 (106.65–146.41)	Neurotoxicity	875 (6.06)	258.6 (240.99–277.5)	Immune effector cell-associated neurotoxicity syndrome	147 (6.01)	886.48 (748.02–1050.57)	Immune effector cell-associated neurotoxicity syndrome	162 (2.93)	420 (358.04–492.68)
Hypotension	32 (2.32)	7.33 (5.16–10.4)	Hypotension	303 (2.10)	6.63 (5.92–7.43)	Death	119 (2.88)	2.06 (1.72–2.48)	Death	514 (3.56)	2.62 (2.4–2.86)	Pyrexia	54 (2.21)	4.02 (3.07–5.27)	Parkinsonism	152 (2.75)	182.07 (154.73–214.24)
Pyrexia	31 (2.25)	4.1 (2.87–5.86)	Hypogammaglobulinaemia	275 (1.91)	221.34 (195.75–250.27)	Pyrexia	86 (2.08)	4.12 (3.32–5.1)	Pyrexia	421 (2.92)	5.36 (4.86–5.91)	Headache	52 (2.13)	2.13 (1.62–2.81)	Neurotoxicity	104 (1.88)	72.46 (59.64–88.04)
Confusional state	18 (1.31)	5.01 (3.15–7.97)	Immune effector cell-associated neurotoxicity syndrome	249 (1.73)	250.67 (220.22–285.33)	Hypotension	81 (1.96)	6.83 (5.48–8.51)	Hypotension	206 (1.43)	4.46 (3.89–5.12)	Decreased appetite	50 (2.04)	5.3 (4.01–7.02)	Malaise	94 (1.70)	2.38 (1.94–2.92)
Aphasia	16 (1.16)	23.31 (14.24–38.16)	Hypoxia	174 (1.21)	22.01 (18.95–25.58)	Confusional state	51 (1.23)	5.76 (4.37–7.6)	Neutropenia	191 (1.32)	6.18 (5.35–7.12)	Neurotoxicity	48 (1.96)	75.38 (56.62–100.36)	Cranial nerve paralysis	90 (1.63)	3890.16 (3038.74–4980.14)
Decreased appetite	15 (1.09)	2.8 (1.68–4.65)	Cytopenia	164 (1.14)	66.55 (56.98–77.73)	Encephalopathy	48 (1.16)	31.04 (23.33–41.29)	Encephalopathy	174 (1.21)	31.03 (26.71–36.06)	Neutropenia	44 (1.80)	8.43 (6.26–11.36)	Facial paralysis	74 (1.34)	58.98 (46.85–74.23)
Mental status changes	12 (0.87)	19.3 (10.93–34.08)	Thrombocytopenia	119 (0.83)	4.67 (3.9–5.59)	Hypoxia	41 (0.99)	17.68 (12.99–24.06)	Confusional state	164 (1.14)	4.35 (3.73–5.08)	Anaemia	41 (1.68)	5.48 (4.02–7.46)	Bell’s palsy	66 (1.19)	578.66 (450.66–743.01)
Tremor	12 (0.87)	3.22 (1.83–5.69)	Neutropenia	111 (0.77)	3.57 (2.96–4.31)	Pancytopenia	39 (0.94)	11.54 (8.42–15.83)	Pancytopenia	150 (1.04)	11.79 (10.04–13.86)	Hypogammaglobulinaemia	29 (1.19)	129.45 (89.66–186.89)	Haemophagocytic lymphohistiocytosis	64 (1.16)	75.51 (58.96–96.71)
Encephalopathy	11 (0.80)	20.31 (11.22–36.77)	Tachycardia	98 (0.68)	4.79 (3.93–5.84)	Tremor	39 (0.94)	4.53 (3.3–6.21)	Aphasia	130 (0.90)	18.1 (15.22–21.52)	Acute kidney injury	28 (1.14)	3.65 (2.52–5.3)	COVID-19	58 (1.05)	3.82 (2.95–4.94)
Hypogammaglobulinaemia	9 (0.65)	70.63 (36.65–136.12)	Confusional state	86 (0.60)	2.27 (1.84–2.81)	Tachycardia	38 (0.92)	7.33 (5.33–10.1)	Tremor	126 (0.87)	3.23 (2.71–3.85)	Hypotension	28 (1.14)	3.57 (2.46–5.18)	Illness	57 (1.03)	7.33 (5.64–9.51)
Memory impairment	9 (0.65)	2.93 (1.52–5.65)	Depressed level of consciousness	69 (0.48)	7.73 (6.1–9.8)	Acute kidney injury	37 (0.89)	2.98 (2.15–4.11)	Thrombocytopenia	104 (0.72)	4.07 (3.35–4.93)	Tremor	27 (1.10)	4.09 (2.8–5.98)	Infection	52 (0.94)	4.17 (3.17–5.48)
Haemophagocytic lymphohistiocytosis	8 (0.58)	37.41 (18.66–74.98)	Haemophagocytic lymphohistiocytosis	67 (0.47)	30.14 (23.69–38.36)	Aphasia	32 (0.77)	19.61 (13.84–27.79)	Hypoxia	99 (0.69)	12.4 (10.17–15.12)	Tachycardia	24 (0.98)	6.92 (4.63–10.35)	Infusion related reaction	44 (0.80)	7.77 (5.78–10.46)
Pleural effusion	8 (0.58)	5.84 (2.92–11.7)	Multiple organ dysfunction syndrome	57 (0.40)	5.44 (4.19–7.06)	Neutropenia	29 (0.70)	2.66 (1.84–3.83)	Tachycardia	94 (0.65)	4.58 (3.74–5.61)	Haemophagocytic lymphohistiocytosis	21 (0.86)	55.56 (36.14–85.42)	Cytopenia	43 (0.78)	44.69 (33.08–60.37)
Infusion related reaction	8 (0.58)	5.66 (2.82–11.34)	Aphasia	54 (0.37)	7.47 (5.72–9.76)	Seizure	27 (0.65)	2.95 (2.02–4.31)	Cytopenia	93 (0.64)	37.17 (30.28–45.63)	Somnolence	21 (0.86)	2.65 (1.73–4.08)	Colitis	42 (0.76)	12.84 (9.48–17.4)
Chills	8 (0.58)	3.06 (1.53–6.13)	Febrile neutropenia	54 (0.37)	3.55 (2.72–4.64)	Infection	27 (0.65)	2.67 (1.83–3.9)	Febrile neutropenia	85 (0.59)	5.59 (4.52–6.92)	Myalgia	20 (0.82)	2.98 (1.92–4.63)	Neutropenia	38 (0.69)	3.18 (2.31–4.38)
Febrile neutropenia	7 (0.51)	4.82 (2.29–10.12)	Encephalopathy	53 (0.37)	9.34 (7.13–12.23)	Haemophagocytic lymphohistiocytosis	26 (0.63)	29.59 (20.1–43.56)	Infection	84 (0.58)	2.57 (2.08–3.19)	Hypoalbuminaemia	19 (0.78)	64.84 (41.26–101.9)	Hypotension	36 (0.65)	2.02 (1.45–2.8)
Hypoxia	6 (0.44)	7.83 (3.51–17.46)	Respiratory failure	53 (0.37)	3.09 (2.36–4.05)	Febrile neutropenia	25 (0.60)	5.13 (3.46–7.6)	Haemophagocytic lymphohistiocytosis	75 (0.52)	33.72 (26.85–42.36)	Cytopenia	19 (0.78)	44.53 (28.34–69.97)	Sepsis	35 (0.63)	3.51 (2.52–4.89)
Depressed level of consciousness	6 (0.44)	7.02 (3.15–15.65)	Staphylococcal infection	46 (0.32)	5.95 (4.46–7.95)	Sepsis	25 (0.60)	3.85 (2.6–5.71)	Sepsis	72 (0.50)	2.76 (2.19–3.48)	Pancytopenia	19 (0.78)	8.77 (5.59–13.78)	Thrombocytopenia	30 (0.54)	3.06 (2.13–4.37)
Septic shock	6 (0.44)	6.33 (2.84–14.11)	Coagulopathy	45 (0.31)	11.6 (8.65–15.55)	Infusion related reaction	21 (0.51)	3.65 (2.38–5.61)	Septic shock	69 (0.48)	6.96 (5.49–8.82)	Chills	18 (0.74)	3.88 (2.44–6.18)	Immune-mediated enterocolitis	27 (0.49)	106.03 (72.49–155.08)
Cardiac arrest	6 (0.44)	3.2 (1.44–7.14)	Pancytopenia	40 (0.28)	3.12 (2.29–4.26)	Brain oedema	20(0.48)	28.73 (18.49–44.63)	Disorientation	57 (0.39)	6.06 (4.67–7.87)	Infusion related reaction	15 (0.61)	5.98 (3.6–9.93)	Respiratory failure	27 (0.49)	4.1 (2.81–5.99)
Atrial fibrillation	6 (0.44)	2.76 (1.24–6.14)	Enterococcal infection	34 (0.24)	30.76 (21.94–43.12)	Mental status changes	20(0.48)	15.13 (9.74–23.49)	Atrial fibrillation	57 (0.39)	2.5 (1.93–3.24)	Thrombocytopenia	14 (0.57)	3.22 (1.91–5.45)	Bacterial infection	26 (0.47)	16.2 (11.02–23.83)
Hypoalbuminaemia	5 (0.36)	30.09 (12.5–72.44)	Rhinovirus infection	33 (0.23)	42.9 (30.42–60.49)	Depressed level of consciousness	20(0.48)	8.88 (5.72–13.79)	Multiple organ dysfunction syndrome	52 (0.36)	4.95 (3.77–6.5)	Confusional state	14 (0.57)	2.18 (1.29–3.68)	Febrile neutropenia	23 (0.42)	3.94 (2.62–5.94)
Tumour lysis syndrome	5 (0.36)	27.07 (11.25–65.16)	Tachypnoea	32 (0.22)	10.4 (7.35–14.72)	Thrombocytopenia	20(0.48)	2.86 (1.84–4.44)	Mental status changes	51 (0.35)	7.8 (5.92–10.27)	Haematotoxicity	13 (0.53)	38.78 (22.47–66.91)	Pancytopenia	22 (0.40)	4.48 (2.94–6.8)
Incontinence	5 (0.36)	21.31 (8.85–51.3)	Nervous system disorder	32 (0.22)	4.19 (2.96–5.92)	Multiple organ dysfunction syndrome	19(0.46)	7.64 (4.86–11.99)	Hypogammaglobulinaemia	49 (0.34)	36.88 (27.82–48.9)	Febrile bone marrow aplasia	12 (0.49)	75.14 (42.57–132.63)	Unevaluable event	21 (0.38)	2.99 (1.95–4.6)
COVID-19 pneumonia	5 (0.36)	18.86 (7.84–45.4)	Bone marrow failure	31 (0.22)	6.24 (4.39–8.89)	Agitation	19(0.46)	5.76 (3.67–9.04)	Pleural effusion	49 (0.34)	3.41 (2.57–4.51)	Sepsis	12 (0.49)	2.71 (1.54–4.79)	Guillain-barre syndrome	20 (0.36)	48.4 (31.17–75.17)
Acute respiratory failure	5 (0.36)	11.93 (4.96–28.72)	*Clostridium difficile* infection	30 (0.21)	6.16 (4.31–8.82)	Respiratory failure	18(0.43)	4.53 (2.85–7.2)	Agitation	48 (0.33)	2.74 (2.06–3.64)	Immunodeficiency	10 (0.41)	18.27 (9.81–34.01)	Influenza	20 (0.36)	2.07 (1.33–3.21)
Pneumonia aspiration	5 (0.36)	9.05 (3.76–21.79)	Disorientation	30 (0.21)	3.19 (2.23–4.56)	Delirium	17(0.41 )	7.85 (4.88–12.65)	Dysgraphia	44 (0.30)	28.25 (20.99–38.02)	Hypoxia	10 (0.41)	7.35 (3.95–13.68)	Immune effector cell-associated hlh-like syndrome	18 (0.33)	5772.29 (3203.84–10399.8)
Hyperkalaemia	5 (0.36)	6.54 (2.72–15.73)	Septic shock	30 (0.21)	3.02 (2.11–4.32)	Cytopenia	16(0.39 )	13.49 (8.25–22.06)	Respiratory failure	43 (0.30)	2.5 (1.85–3.37)	Febrile neutropenia	9 (0.37)	3.48 (1.81–6.71)	Septic shock	17 (0.31)	4.46 (2.77–7.18)

Unevaluable event was retained as a nonspecific but formally coded MedDRA PT, that met the predefined signal criteria; it was interpreted descriptively and was not used to define product-level profile labels.

Across the six products, cytokine release syndrome was consistently among the leading PT-level signals, indicating a recurrent CAR-T class-related reporting pattern. Other repeatedly observed signals included immune effector cell-associated neurotoxicity syndrome, neurotoxicity, hypotension, pyrexia, and several infection- and hematologic-related PTs.

Product-level differences were also observed. Yescarta and Tecartus showed higher reporting proportions of immune effector cell-associated neurotoxicity syndrome, with 1207 reports (8.36%) for Yescarta and 439 reports (10.61%) for Tecartus. Neurotoxicity was also more frequently reported for Yescarta [875 reports (6.06%)] than for Kymriah [309 reports (2.14%)] or Carvykti [104 reports (1.88%)]. Kymriah was more strongly represented by infection-, immune-, and hematologic-related PTs, including hypogammaglobulinaemia [275 reports (1.91%)], hypoxia [174 reports (1.21%)], and neutropenia [111 reports (0.77%)]. Carvykti showed several neurologic PTs, including Parkinsonism [152 reports (2.75%)], cranial nerve paralysis [90 reports (1.63%)], facial paralysis [74 reports (1.34%)], and Bell’s palsy [66 reports (1.19%)]. In Abecma, systemic symptom-related PTs ranked relatively high, including fatigue [151 reports (6.17%)], headache [52 reports (2.13%)], and decreased appetite [50 reports (2.04%)].

The concise product-level profile labels shown in Figure 1 were derived from the integrated interpretation of clinically interpretable eligible PT-level signals, top-ranked clinically interpretable PT signals, SOC-level distributions, and cross-product overlap patterns. These labels were used as descriptive summaries of reporting-signal patterns rather than as incidence-based or causality-based toxicity phenotypes.

### 3.3 SOC-level distribution of eligible PT signals

Table 4 summarizes the SOC-level distribution of eligible PT-level positive AE signals for the six CAR-T products. The percentages in Table 4 represent the proportion of eligible PT signals assigned to each SOC within a given product, rather than the incidence or reporting frequency of adverse events. Overall, nervous system disorders and infections and infestations were among the most frequently represented SOC categories, but their relative contributions differed across products.

**TABLE 4:** SOC-level distribution of eligible PT-level positive AE signals for six CAR-T products.

SOC	Breyanzi n (%)	Kymriah n (%)	Tecartus n (%)	Yescarta n (%)	Abecma n (%)	Carvykti n (%)
Nervous system disorders	7 (17.9)	22 (15.6)	16 (19.8)	25 (16.8)	10 (17.5)	31 (29.2)
Infections and infestations	6 (15.4)	47 (33.3)	15 (18.5)	45 (30.2)	9 (15.8)	29 (27.4)
Metabolism and nutrition disorders	6(15.4)	7(5.0)	9(11.1)	7(4.7)	5(8.8)	4(3.8)
Blood and lymphatic system disorders	4(10.3)	14(9.9)	9(11.1)	13(8.7)	11(19.3)	7(6.6)
Respiratory, thoracic and mediastinal disorders	4(10.3)	9(6.4)	7(8.6)	9(6.0)	4(7.0)	5(4.7)
Immune system disorders	3(7.7)	8(5.7)	5(6.2)	6(4.0)	4(7.0)	8(7.5)
General disorders and administration site conditions	2(5.1)	5(3.5)	4(4.9)	5(3.4)	3(5.3)	5 (4.7)
Psychiatric disorders	2(5.1)	4(2.8)	5(6.2)	6(4.0)	2(3.5)	3(2.8)
Cardiac disorders	2(5.1)	5(3.5)	4(4.9)	9(6.0)	2(3.5)	0(0)
Vascular disorders	1(2.6)	6(4.3)	2(2.5)	6(4.0)	1(1.8)	1(0.9)
Renal and urinary disorders	1(2.6)	3(2.1)	3(3.7)	2(1.3)	2(3.5)	0(0)
Injury, poisoning and procedural complications	1(2.6)	1(0.7)	1(1.2)	3(2.0)	1(1.8)	2(1.9)
Gastrointestinal disorders	0(0)	2(1.4)	0(0)	7(4.7)	0(0)	9(8.5)
Hepatobiliary disorders	0(0)	3(2.1)	1(1.2)	2(1.3)	2(3.5)	1(0.9)
Eye disorders	0(0)	3(2.1)	0(0)	2(1.3)	0(0)	1(0.9)
Congenital, familial and genetic disorders	0(0)	1(0.7)	0(0)	1(0.7)	0(0)	0(0)
Skin and subcutaneous tissue disorders	0(0)	1(0.7)	0(0)	0(0)	0(0)	0(0)
Musculoskeletal and connective tissue disorders	0(0)	0(0)	0(0)	0(0)	1(1.8)	0(0)
Endocrine disorders	0(0)	0(0)	0(0)	1(0.7)	0(0)	0(0)

Percentages were calculated as the number of eligible PT-level signals assigned to a given SOC, divided by the total number of eligible PT-level signals for that product.SOC, percentages reflect the distribution of eligible positive PT, signals across SOCs, and should not be interpreted as event frequencies or incidence rates.

Carvykti showed the highest proportion of signals assigned to nervous system disorders (29.2%), followed by Tecartus (19.8%), Breyanzi (17.9%), Abecma (17.5%), Yescarta (16.8%), and Kymriah (15.6%), indicating a relatively stronger neurologic signal concentration for Carvykti at the SOC level. For infections and infestations, Kymriah, Yescarta, and Carvykti showed higher proportions, accounting for 33.3%, 30.2%, and 27.4% of eligible PT signals, respectively, compared with Tecartus (18.5%), Abecma (15.8%), and Breyanzi (15.4%).

Other SOC categories also contributed to product-skewed patterns. Abecma had the highest proportion of blood and lymphatic system disorder signals (19.3%), compared with Tecartus (11.1%), Breyanzi (10.3%), Kymriah (9.9%), Yescarta (8.7%), and Carvykti (6.6%). Breyanzi showed comparable proportions of infections and infestations and metabolism and nutrition disorders (both 15.4%), whereas Carvykti also showed a relatively higher proportion of gastrointestinal disorder signals (8.5%) than the other products. These findings indicate that the SOC-level signal distribution was not uniform across CAR-T products and supported the product-skewed reporting patterns observed in the PT-level analyses.

### 3.4 Comparison of positive PT signals among different products

Figure 4 presents the ROR forest plot for the top 30 PT-level positive AE signals associated with Carvykti. Carvykti was selected for main-text presentation because it showed the highest proportion of nervous-system SOC signals and several distinctive neurologic PT-level signals. Forest plots for Breyanzi, Kymriah, Tecartus, Yescarta, and Abecma are provided in Supplementary Figures S1–S5.

**FIGURE 4:**
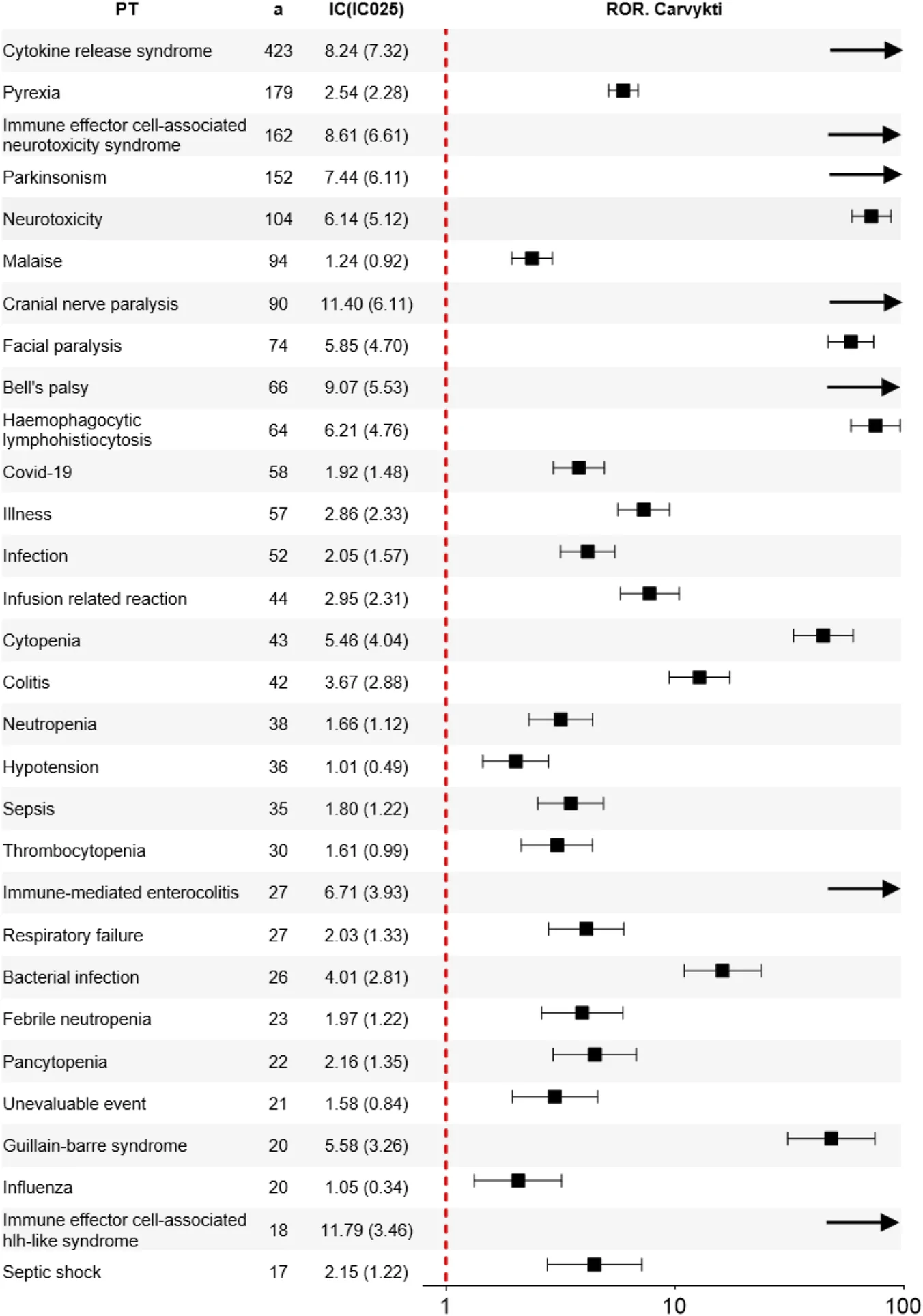
Forest plot of RORs (95% CI) for the top 30 PT-level positive AE signals associated with Carvykti. Unevaluable event was retained as a nonspecific but formally coded MedDRA PT and was interpreted descriptively.

Figure 5 shows the overlap and product-skewed distribution of the top 30 PT-level positive AE signals across the six CAR-T products. In this network, shared PTs were linked to multiple products, whereas product-skewed PTs were mainly connected to one product or a limited number of products. CRS, immune effector cell-associated neurotoxicity syndrome, neurotoxicity, hypotension, pyrexia, and several infection- and hematologic-related PTs formed the recurrent CAR-T class-related signal pattern.

**FIGURE 5:**
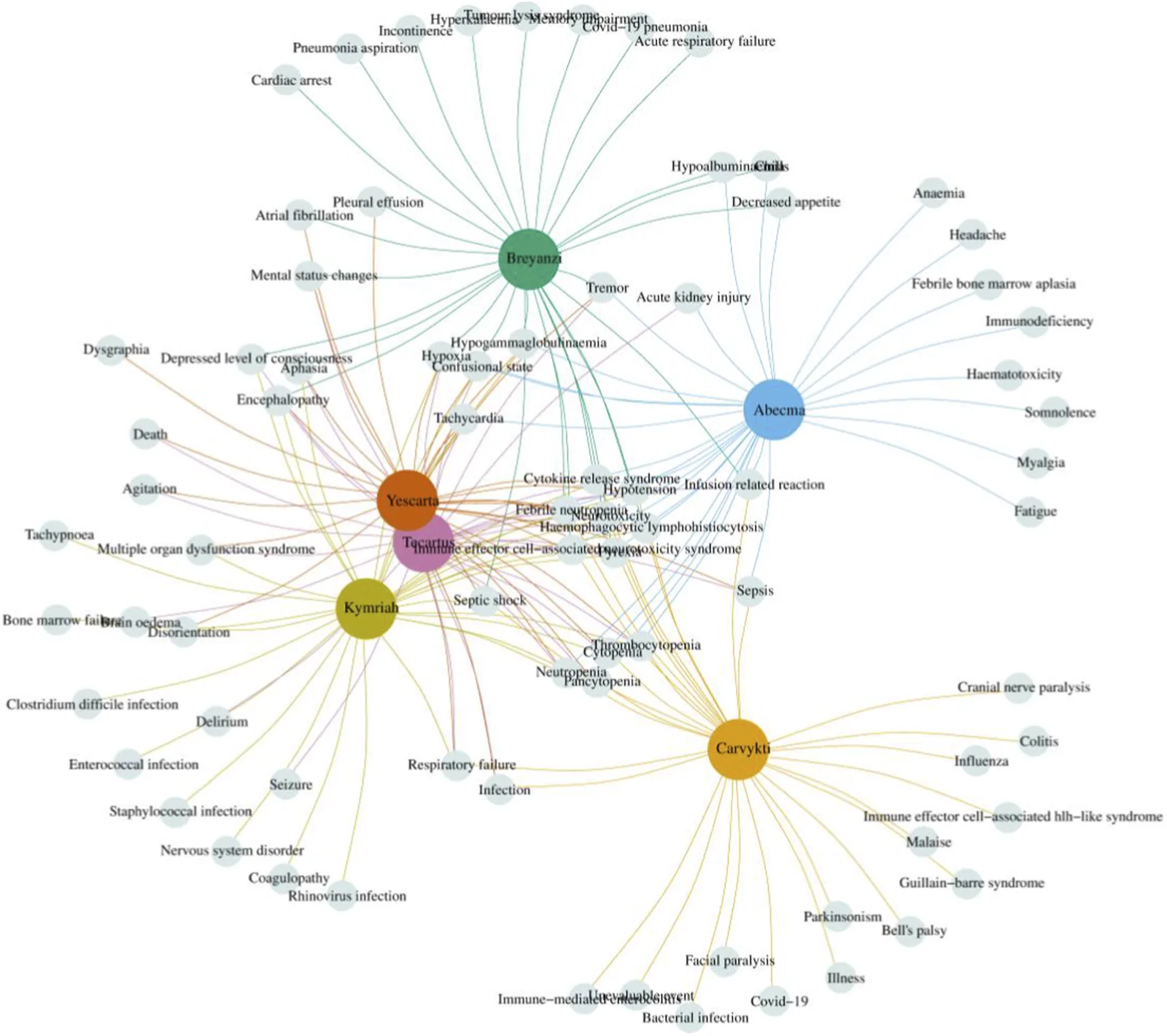
Network diagram of the top 30 PT-level positive AE signals associated with six CAR-T products in the FAERS database. The diagram illustrates shared and product-skewed PT-level signals among different CAR-T products.

Product-skewed patterns were also evident. Breyanzi showed a relatively balanced reporting profile without a single dominant product-skewed category. Kymriah was more strongly represented by infection- and immunosuppression-related PTs, including hypogammaglobulinaemia, hypoxia, and neutropenia. Tecartus showed an ICANS-focused profile, whereas Yescarta showed an acute neurotoxicity-skewed reporting profile. Abecma was characterized by cytopenia/fatigue-related reporting, supported by blood and lymphatic system signals and high-ranking fatigue-related systemic symptoms. Carvykti was mainly characterized by a delayed/atypical neurologic reporting profile, including Parkinsonism, cranial nerve paralysis, facial paralysis, and Bell’s palsy.

### 3.5 Subgroup analyses

#### 3.5.1 Age-subgroup analysis

Age-stratified analyses showed heterogeneous reporting patterns across the six CAR-T products (Supplementary Figure S6). Breyanzi showed a positive signal in patients aged 65–85 years (ROR 1.42, 95% CI 1.13–1.78), whereas Kymriah showed a positive signal in patients aged <18 years (ROR 1.40, 95% CI 1.29–1.52). No consistent age-related pattern was observed for the other products. Missing-age reports were retained as a separate subgroup, and estimates based on small subgroup counts should be interpreted cautiously.

#### 3.5.2 Sex-subgroup analysis

Sex-stratified analyses showed generally modest differences between male and female subgroups across the six CAR-T products (Supplementary Figure S7). ROR estimates were broadly comparable between sexes for Breyanzi, Kymriah, Abecma, and Carvykti, whereas lower RORs were observed for Tecartus in males and for Yescarta in both sexes. Missing-sex reports were retained as a separate subgroup and showed higher ROR estimates for Tecartus and Yescarta. Overall, no consistent sex-specific reporting pattern was observed across products.

#### 3.5.3 Outcome-subgroup analysis

Outcome-subgroup analysis showed heterogeneous reporting patterns across CAR-T products (Supplementary Figure S8). Positive signals were observed for hospitalization with Breyanzi (ROR 1.72, 95% CI 1.37–2.17), and for the “Other” outcome category with Kymriah (ROR 1.38, 95% CI 1.28–1.50), Tecartus (ROR 1.34, 95% CI 1.15–1.56), and Abecma (ROR 2.17, 95% CI 1.80–2.61). Carvykti showed positive signals for serious outcomes (ROR 1.22, 95% CI 1.09–1.37) and hospitalization (ROR 1.46, 95% CI 1.29–1.66). No corresponding positive outcome signal was observed for Yescarta.

Collectively, subgroup analyses showed variation in disproportionality patterns across age, sex, and clinical outcome categories. Age-related and outcome-specific variations were observed for selected products, whereas sex-related differences were generally limited.

## 4 Discussion

In this FAERS-based pharmacovigilance analysis, six CAR-T products showed a recurrent adverse-event reporting pattern centered on CRS, ICANS/neurotoxicity, hypotension, pyrexia, infection-related events, and hematologic events. This pattern is consistent with the established safety profile of CAR-T therapy, in which excessive immune activation, neurologic involvement, infection susceptibility, and myelosuppression represent major clinical concerns (Morris et al., 2022; Schubert et al., 2021; Lee et al., 2019; Shahid et al., 2024; Hill and Seo, 2020). Because FAERS is a spontaneous reporting system, these findings should be interpreted as reporting signals rather than incidence estimates or evidence of causality (Cutroneo et al., 2024).

Despite this shared reporting backbone, product-level differences were observed. Yescarta and Tecartus showed a higher concentration of neurotoxicity-related PTs, including ICANS and neurotoxicity. This finding is consistent with the recognized importance of neurologic toxicity in CAR-T therapy and with previous pharmacovigilance evidence suggesting that AE reporting patterns may differ among CAR-T products (Morris et al., 2022; Schubert et al., 2021; Lee et al., 2019; Guo et al., 2024; Liu et al., 2026). However, the present FAERS-based analysis cannot determine whether these differences reflect intrinsic product biology, differences in indications, patient characteristics, treatment settings, or reporting behavior.

Kymriah showed a reporting pattern more strongly represented by infection-, immune-, and hematologic-related PTs, including hypogammaglobulinaemia, hypoxia, and neutropenia. These findings are clinically plausible because infection risk after CAR-T therapy may be influenced by immune reconstitution, B-cell aplasia, hypogammaglobulinaemia, cytopenia, prior therapies, and disease-related immunosuppression (Schubert et al., 2021; Shahid et al., 2024; Hill and Seo, 2020). Therefore, the Kymriah-associated pattern observed in this study should be interpreted as a signal of relatively prominent infection- and immunosuppression-related reporting, rather than as a direct comparison of true infection incidence across products.

Carvykti showed several neurologic PTs that were less typical of conventional acute ICANS, including Parkinsonism, cranial nerve paralysis, facial paralysis, and Bell’s palsy. These findings are consistent with published concerns regarding atypical neurologic events after BCMA-directed CAR-T therapy (Berdeja et al., 2021; Van Oekelen et al., 2021; Karschnia et al., 2023). This pattern is also consistent with recent real-world evidence showing more frequent delayed neurotoxicity with cilta-cel than with ide-cel, while conventional ICANS did not show a similar difference (Hansen et al., 2025). The present FAERS findings support an atypical neurologic reporting pattern for Carvykti that is broadly consistent with the delayed neurologic toxicities described in previous clinical studies. Accordingly, Figure 1 label of delayed neurotoxicity was used as a descriptive shorthand for this delayed/atypical neurologic reporting profile, supported by the observed signal pattern and prior clinical literature. However, because FAERS reports do not provide uniform time-to-onset information, this label should not be interpreted as direct evidence that delayed onset was established in the present dataset. Further studies with clinical timing, exposure denominators, and standardized neurologic assessment are needed to clarify whether these signals represent delayed neurologic toxicity and to define their clinical risk factors.

The observed product-skewed reporting patterns may be related to differences in target antigen, co-stimulatory domain, expansion kinetics, *in vivo* persistence, disease indication, prior treatment exposure, and clinical monitoring practices (Abramson et al., 2020; Schuster et al., 2019; Wang et al., 2020; Neelapu et al., 2017; Munshi et al., 2021; Berdeja et al., 2021; Morris et al., 2022; Schubert et al., 2021). Patient composition may further contribute to this heterogeneity, as CAR-T products are used across pediatric and adult populations and across different underlying malignancies, particularly lymphoma and multiple myeloma, with differences in disease characteristics, prior treatment exposure, and treatment line. For example, differences between CD28^−^and 4-1BB-based CAR constructs may affect expansion and persistence profiles, which could contribute to heterogeneity in toxicity reporting. Nevertheless, FAERS data alone cannot separate biological mechanisms from confounding by indication, patient population, or reporting bias. Therefore, these product-level differences should be regarded as pharmacovigilance hypotheses that require validation in clinical cohorts.

From a clinical perspective, these findings suggest that CAR-T safety management should combine standardized prevention of class-related toxicities with product-aware monitoring. CRS and ICANS remain core toxicities requiring early recognition and standardized grading (Schubert et al., 2021; Lee et al., 2019). For products with prominent neurotoxicity-related reporting, dynamic neurologic assessment and ICANS-oriented monitoring should be emphasized (Lee et al., 2019; Van Oekelen et al., 2021; Karschnia et al., 2023). For products with higher infection- and immunosuppression-related reporting, infection surveillance, antimicrobial prophylaxis when appropriate, and immunoglobulin monitoring or replacement should be considered according to clinical guidelines and patient-specific risk factors (Shahid et al., 2024; Hill and Seo, 2020). These recommendations should be interpreted as signal-informed considerations for clinical surveillance rather than evidence of comparative safety or causal differences among CAR-T products. Product-aware monitoring should complement, rather than replace, established guideline-based management and individualized patient assessment.

Several limitations should be considered when interpreting these findings. As a spontaneous reporting system, FAERS is subject to under-reporting, stimulated reporting, potential residual duplicate reporting, and other reporting biases, which may also vary across countries and regions. Cross-product comparisons may also be confounded by differences in approved indications, patient populations, disease stage, prior and concomitant therapies, manufacturing platforms, year of approval, and duration of post-marketing exposure, while incomplete clinical information limits adjustment for these factors. Moreover, the lack of reliable exposure denominators precludes estimation of true incidence, and disproportionality signals reflect reporting associations rather than causal relationships or direct measures of clinical risk. Therefore, observed differences across CAR-T products should be interpreted as differences in post-marketing reporting patterns rather than as evidence of differences in intrinsic product toxicity. Despite predefined PT-exclusion rules, residual misclassification may remain, and the limited post-marketing data for Aucatzyl precluded its inclusion in the PT- and SOC-level analyses. More broadly, evidence across different phases of clinical trials underscores the complementary role of post-marketing pharmacovigilance in identifying uncommon, delayed, or incompletely characterized adverse events that may not be fully captured during pre-approval clinical development (Upadhye et al., 2025).

Future studies should validate FAERS-derived signals using independent pharmacovigilance databases such as VigiBase and EudraVigilance, together with longitudinal and machine-learning approaches. Integration with active surveillance systems and clinically characterized real-world registries may further strengthen the clinical interpretation and translational relevance of CAR-T safety signals.

## 5 Conclusion

In this FAERS-based pharmacovigilance analysis, six CAR-T products shared a recurrent reporting pattern centered on CRS, ICANS/neurotoxicity, hypotension, pyrexia, infection-related events, and hematologic events. At the same time, the distribution of positive signals varied across products. Breyanzi showed a relatively mixed reporting pattern, whereas infection- and immunosuppression-related signals were relatively prominent for Kymriah, ICANS and other neurologic signals for Tecartus and Yescarta, cytopenia- and fatigue-related signals for Abecma, and several neurologic signals, including Parkinsonism and cranial nerve-related events, for Carvykti. These findings represent product-associated disproportionality patterns rather than differences in clinical incidence or intrinsic toxicity and do not establish causal relationships. Further validation using prospective real-world studies with reliable exposure denominators and clinical time-to-event information is warranted.

## Data Availability

The original contributions presented in the study are included in the article/Supplementary Material, further inquiries can be directed to the corresponding authors.
