# Supplementary material for: Comparative real-world pharmacovigilance analysis of adverse event signals associated with six approved CAR-T products using the FAERS database
Source: Front Pharmacol. 2026 Oct 7;17:1913235. doi: 10.3389/fphar.2026.1913235 (PMC13683057; doi:10.3389/fphar.2026.1913235)
Supplement: Supplementary file 1 [file Supplementaryfile1.docx]

**Comparative Real-World Pharmacovigilance Analysis of Adverse Event Signals Associated with Six Approved CAR-T Products Using the FAERS Database**

Shengyuan Xiao^1,2^, Ying Li^1,2^, Hui Wang^1,2*^ , Longcai Tong^1^, Rong Li^1,2^ ^*^

^1^. Hunan Provincial Key Laboratory of Basic and Clinical Pharmacological Research of Gastrointestinal Cancer, Department of Pharmacy, Institute of Pharmacy and Pharmacology, the Second Affiliated Hospital, University of South China, Hengyang, 421001, Hunan, China

^2^. Hengyang Medical School, University of South China, Hengyang, 421001, Hunan, China

*Corresponding authors:

[wanghuiwh828@126.com (Hui Wang);](mailto:Wanghuiwh828@126.com;) l7979r@163.com (Rong Li)

**Table S1** PT and SOC for the positive signal detection of Breyanzi in the FAERS database

| PT | SOC | PRR | n | IC | IC025 |
| --- | --- | --- | --- | --- | --- |
| Cytokine release syndrome | Immune system disorders | 625.97 | 215 | 9.27 | 7.11 |
| Immune effector cell-associated neurotoxicity syndrome | Nervous system disorders | 1054.87 | 106 | 10 | 6.31 |
| Neurotoxicity | Nervous system disorders | 230.24 | 84 | 7.84 | 5.63 |
| Hypotension | Vascular disorders | 7.18 | 32 | 2.84 | 2.09 |
| Pyrexia | General disorders and administration site conditions | 4.03 | 31 | 2.01 | 1.36 |
| Confusional state | Psychiatric disorders | 4.96 | 18 | 2.31 | 1.37 |
| Aphasia | Nervous system disorders | 23.05 | 16 | 4.53 | 2.62 |
| Decreased appetite | Metabolism and nutrition disorders | 2.78 | 15 | 1.47 | 0.6 |
| Mental status changes | Psychiatric disorders | 19.14 | 12 | 4.26 | 2.19 |
| Tremor | Nervous system disorders | 3.2 | 12 | 1.68 | 0.65 |
| Encephalopathy | Nervous system disorders | 20.16 | 11 | 4.33 | 2.12 |
| Hypogammaglobulinaemia | Immune system disorders | 70.18 | 9 | 6.13 | 2.23 |
| Memory impairment | Nervous system disorders | 2.92 | 9 | 1.55 | 0.38 |
| Haemophagocytic lymphohistiocytosis | Immune system disorders | 37.2 | 8 | 5.22 | 1.92 |
| Pleural effusion | Respiratory, thoracic and mediastinal disorders | 5.81 | 8 | 2.54 | 0.96 |
| Infusion related reaction | Injury, poisoning and procedural complications | 5.63 | 8 | 2.49 | 0.93 |
| Chills | General disorders and administration site conditions | 3.05 | 8 | 1.61 | 0.35 |
| Febrile neutropenia | Blood and lymphatic system disorders | 4.8 | 7 | 2.26 | 0.68 |
| Hypoxia | Respiratory, thoracic and mediastinal disorders | 7.8 | 6 | 2.96 | 0.89 |
| Depressed level of consciousness | Nervous system disorders | 6.99 | 6 | 2.81 | 0.82 |
| Septic shock | Infections and infestations | 6.31 | 6 | 2.66 | 0.75 |
| Cardiac arrest | Cardiac disorders | 3.19 | 6 | 1.67 | 0.19 |
| Atrial fibrillation | Cardiac disorders | 2.75 | 6 | 1.46 | 0.04 |
| Hypoalbuminaemia | Metabolism and nutrition disorders | 29.99 | 5 | 4.91 | 1.18 |
| Tumour lysis syndrome | Metabolism and nutrition disorders | 26.97 | 5 | 4.75 | 1.16 |
| Incontinence | Renal and urinary disorders | 21.24 | 5 | 4.41 | 1.1 |
| Covid-19 pneumonia | Infections and infestations | 18.8 | 5 | 4.23 | 1.06 |
| Acute respiratory failure | Respiratory, thoracic and mediastinal disorders | 11.89 | 5 | 3.57 | 0.9 |
| Pneumonia aspiration | Infections and infestations | 9.02 | 5 | 3.17 | 0.77 |
| Hyperkalaemia | Metabolism and nutrition disorders | 6.52 | 5 | 2.7 | 0.58 |
| Pancytopenia | Blood and lymphatic system disorders | 4.07 | 5 | 2.02 | 0.25 |
| Cytopenia | Blood and lymphatic system disorders | 16.49 | 4 | 4.04 | 0.72 |
| Cytomegalovirus infection | Infections and infestations | 10.96 | 4 | 3.45 | 0.58 |
| Hyperglycaemia | Metabolism and nutrition disorders | 4.87 | 4 | 2.28 | 0.16 |
| Organising pneumonia | Respiratory, thoracic and mediastinal disorders | 25.64 | 3 | 4.68 | 0.39 |
| Encephalitis | Infections and infestations | 19.84 | 3 | 4.31 | 0.35 |
| Haematotoxicity | Blood and lymphatic system disorders | 15.78 | 3 | 3.98 | 0.3 |
| Bacteraemia | Infections and infestations | 11.73 | 3 | 3.55 | 0.23 |
| Hypervolaemia | Metabolism and nutrition disorders | 6.8 | 3 | 2.77 | 0.03 |

**Table S2** PT and SOC for the positive signal detection of Kymriah in the FAERS database

| PT | SOC | PRR | n | IC | IC025 |
| --- | --- | --- | --- | --- | --- |
| Cytokine release syndrome | Immune system disorders | 323.79 | 1084 | 8.21 | 7.77 |
| Pyrexia | General disorders and administration site conditions | 9.43 | 756 | 3.23 | 3.11 |
| Neurotoxicity | Nervous system disorders | 82.35 | 309 | 6.33 | 5.83 |
| Hypotension | Vascular disorders | 6.51 | 303 | 2.7 | 2.51 |
| Hypogammaglobulinaemia | Immune system disorders | 217.13 | 275 | 7.68 | 6.7 |
| Immune effector cell-associated neurotoxicity syndrome | Nervous system disorders | 246.36 | 249 | 7.85 | 6.72 |
| Hypoxia | Respiratory, thoracic and mediastinal disorders | 21.76 | 174 | 4.44 | 4.05 |
| Cytopenia | Blood and lymphatic system disorders | 65.8 | 164 | 6.01 | 5.32 |
| Thrombocytopenia | Blood and lymphatic system disorders | 4.64 | 119 | 2.21 | 1.9 |
| Neutropenia | Blood and lymphatic system disorders | 3.55 | 111 | 1.83 | 1.52 |
| Tachycardia | Cardiac disorders | 4.76 | 98 | 2.25 | 1.91 |
| Confusional state | Psychiatric disorders | 2.27 | 86 | 1.18 | 0.85 |
| Depressed level of consciousness | Nervous system disorders | 7.7 | 69 | 2.94 | 2.46 |
| Haemophagocytic lymphohistiocytosis | Immune system disorders | 30.01 | 67 | 4.9 | 4.03 |
| Multiple organ dysfunction syndrome | General disorders and administration site conditions | 5.42 | 57 | 2.44 | 1.95 |
| Aphasia | Nervous system disorders | 7.45 | 54 | 2.89 | 2.34 |
| Febrile neutropenia | Blood and lymphatic system disorders | 3.54 | 54 | 1.82 | 1.37 |
| Encephalopathy | Nervous system disorders | 9.31 | 53 | 3.21 | 2.61 |
| Respiratory failure | Respiratory, thoracic and mediastinal disorders | 3.08 | 53 | 1.62 | 1.17 |
| Staphylococcal infection | Infections and infestations | 5.94 | 46 | 2.57 | 2 |
| Coagulopathy | Blood and lymphatic system disorders | 11.56 | 45 | 3.53 | 2.8 |
| Pancytopenia | Blood and lymphatic system disorders | 3.11 | 40 | 1.64 | 1.11 |
| Enterococcal infection | Infections and infestations | 30.69 | 34 | 4.93 | 3.56 |
| Rhinovirus infection | Infections and infestations | 42.8 | 33 | 5.4 | 3.76 |
| Tachypnoea | Respiratory, thoracic and mediastinal disorders | 10.38 | 32 | 3.37 | 2.51 |
| Nervous system disorder | Nervous system disorders | 4.18 | 32 | 2.06 | 1.43 |
| Bone marrow failure | Blood and lymphatic system disorders | 6.23 | 31 | 2.64 | 1.91 |
| Clostridium difficile infection | Infections and infestations | 6.15 | 30 | 2.62 | 1.88 |
| Disorientation | Psychiatric disorders | 3.18 | 30 | 1.67 | 1.05 |
| Septic shock | Infections and infestations | 3.02 | 30 | 1.59 | 0.98 |
| Disseminated intravascular coagulation | Blood and lymphatic system disorders | 8.71 | 29 | 3.12 | 2.26 |
| Mental status changes | Psychiatric disorders | 4.43 | 29 | 2.14 | 1.46 |
| Fungal infection | Infections and infestations | 3.64 | 28 | 1.86 | 1.2 |
| Tumour lysis syndrome | Metabolism and nutrition disorders | 13.45 | 26 | 3.74 | 2.64 |
| Hemiparesis | Nervous system disorders | 6.5 | 26 | 2.7 | 1.87 |
| Cytomegalovirus infection | Infections and infestations | 6.56 | 25 | 2.71 | 1.87 |
| Candida infection | Infections and infestations | 5.3 | 25 | 2.41 | 1.62 |
| Delirium | Psychiatric disorders | 2.88 | 23 | 1.52 | 0.83 |
| Motor dysfunction | Nervous system disorders | 9.42 | 22 | 3.23 | 2.18 |
| Paraparesis | Nervous system disorders | 50.69 | 21 | 5.64 | 3.33 |
| Organ failure | General disorders and administration site conditions | 20.06 | 20 | 4.32 | 2.76 |
| Respiratory distress | Respiratory, thoracic and mediastinal disorders | 3.08 | 20 | 1.62 | 0.86 |
| Pseudomonas infection | Infections and infestations | 9.62 | 18 | 3.26 | 2.06 |
| Clostridium difficile colitis | Infections and infestations | 7.19 | 18 | 2.84 | 1.77 |
| Brain oedema | Nervous system disorders | 6.18 | 18 | 2.63 | 1.61 |
| Acute respiratory distress syndrome | Respiratory, thoracic and mediastinal disorders | 4.35 | 18 | 2.12 | 1.22 |
| Lymphadenopathy | Blood and lymphatic system disorders | 2.19 | 18 | 1.13 | 0.38 |
| Dysgraphia | Nervous system disorders | 10.85 | 17 | 3.44 | 2.13 |
| Bacterial infection | Infections and infestations | 4.05 | 17 | 2.02 | 1.11 |
| Acute respiratory failure | Respiratory, thoracic and mediastinal disorders | 3.87 | 17 | 1.95 | 1.06 |
| Hepatic failure | Hepatobiliary disorders | 2.42 | 17 | 1.27 | 0.48 |
| Viral sinusitis | Infections and infestations | 379.5 | 16 | 8.42 | 3.29 |
| Bk virus infection | Infections and infestations | 21.2 | 16 | 4.4 | 2.57 |
| Adenovirus infection | Infections and infestations | 24.02 | 15 | 4.58 | 2.57 |
| Aspergillus infection | Infections and infestations | 8.59 | 15 | 3.1 | 1.82 |
| Hypervolaemia | Metabolism and nutrition disorders | 3.25 | 15 | 1.7 | 0.79 |
| Hepatotoxicity | Hepatobiliary disorders | 2.99 | 15 | 1.58 | 0.69 |
| Human herpesvirus 6 infection | Infections and infestations | 32.66 | 14 | 5.02 | 2.64 |
| Pulmonary toxicity | Respiratory, thoracic and mediastinal disorders | 9.23 | 14 | 3.2 | 1.83 |
| Klebsiella infection | Infections and infestations | 11.54 | 13 | 3.52 | 1.94 |
| Graft versus host disease | Immune system disorders | 7.68 | 13 | 2.94 | 1.6 |
| Capillary leak syndrome | Vascular disorders | 26.83 | 12 | 4.74 | 2.36 |
| Gastrointestinal infection | Infections and infestations | 5.93 | 12 | 2.57 | 1.3 |
| Facial paralysis | Nervous system disorders | 3.6 | 12 | 1.85 | 0.78 |
| Generalised tonic-clonic seizure | Nervous system disorders | 2.03 | 12 | 1.02 | 0.11 |
| Hypofibrinogenaemia | Blood and lymphatic system disorders | 49.1 | 11 | 5.6 | 2.45 |
| Viral upper respiratory tract infection | Infections and infestations | 9.08 | 11 | 3.18 | 1.6 |
| Escherichia infection | Infections and infestations | 6.01 | 11 | 2.59 | 1.25 |
| Nephropathy toxic | Renal and urinary disorders | 4.38 | 11 | 2.13 | 0.94 |
| Bacteraemia | Infections and infestations | 4.11 | 11 | 2.04 | 0.87 |
| Bone marrow disorder | Blood and lymphatic system disorders | 14.27 | 10 | 3.83 | 1.82 |
| Leukoencephalopathy | Nervous system disorders | 11.33 | 10 | 3.5 | 1.67 |
| Acute graft versus host disease | Immune system disorders | 9.09 | 10 | 3.18 | 1.52 |
| Neurological symptom | Nervous system disorders | 7.66 | 10 | 2.93 | 1.38 |
| Pulmonary haemorrhage | Respiratory, thoracic and mediastinal disorders | 5.35 | 10 | 2.42 | 1.07 |
| Status epilepticus | Nervous system disorders | 3.84 | 10 | 1.94 | 0.74 |
| Respiratory syncytial virus infection | Infections and infestations | 4.74 | 9 | 2.24 | 0.87 |
| Malnutrition | Metabolism and nutrition disorders | 3.54 | 9 | 1.82 | 0.58 |
| Sinus tachycardia | Cardiac disorders | 2.72 | 9 | 1.44 | 0.3 |
| Cytokine storm | Immune system disorders | 52.42 | 8 | 5.69 | 1.99 |
| Enterovirus infection | Infections and infestations | 44.68 | 8 | 5.46 | 1.96 |
| Neurological decompensation | Nervous system disorders | 15.27 | 8 | 3.93 | 1.6 |
| Gastrointestinal toxicity | Gastrointestinal disorders | 8.06 | 8 | 3.01 | 1.21 |
| Concomitant disease aggravated | General disorders and administration site conditions | 5.48 | 8 | 2.45 | 0.91 |
| Bronchopulmonary aspergillosis | Infections and infestations | 4.52 | 8 | 2.17 | 0.74 |
| Cardiotoxicity | Cardiac disorders | 4.1 | 8 | 2.03 | 0.64 |
| Systemic candida | Infections and infestations | 12.9 | 7 | 3.68 | 1.35 |
| Staphylococcal bacteraemia | Infections and infestations | 7.24 | 7 | 2.85 | 1 |
| Organising pneumonia | Respiratory, thoracic and mediastinal disorders | 5.73 | 7 | 2.52 | 0.83 |
| Haemodynamic instability | Vascular disorders | 4.16 | 7 | 2.05 | 0.55 |
| Coronavirus infection | Infections and infestations | 4.01 | 7 | 2 | 0.52 |
| Hepatomegaly | Hepatobiliary disorders | 3.03 | 7 | 1.6 | 0.25 |
| Electrolyte imbalance | Metabolism and nutrition disorders | 2.71 | 7 | 1.44 | 0.14 |
| Cardiorenal syndrome | Cardiac disorders | 46.11 | 6 | 5.51 | 1.53 |
| Alpha haemolytic streptococcal infection | Infections and infestations | 29.24 | 6 | 4.86 | 1.44 |
| Lymphocytosis | Blood and lymphatic system disorders | 12.03 | 6 | 3.58 | 1.13 |
| Congenital aplasia | Congenital, familial and genetic disorders | 11.01 | 6 | 3.46 | 1.09 |
| Pulseless electrical activity | Cardiac disorders | 5.22 | 6 | 2.38 | 0.61 |
| Oliguria | Renal and urinary disorders | 4.18 | 6 | 2.06 | 0.43 |
| Encephalitis | Infections and infestations | 3.8 | 6 | 1.92 | 0.35 |
| Hypoalbuminaemia | Metabolism and nutrition disorders | 3.44 | 6 | 1.78 | 0.26 |
| Shock haemorrhagic | Vascular disorders | 3.24 | 6 | 1.69 | 0.2 |
| Purpura | Skin and subcutaneous tissue disorders | 2.91 | 6 | 1.54 | 0.1 |
| Stenotrophomonas infection | Infections and infestations | 16.59 | 5 | 4.05 | 1.02 |
| Venoocclusive disease | Vascular disorders | 8.14 | 5 | 3.02 | 0.71 |
| Chronic graft versus host disease | Immune system disorders | 5.84 | 5 | 2.54 | 0.51 |
| Hypernatraemia | Metabolism and nutrition disorders | 4.34 | 5 | 2.12 | 0.3 |
| Herpes simplex | Infections and infestations | 3.36 | 5 | 1.75 | 0.09 |
| Corynebacterium infection | Infections and infestations | 36.77 | 4 | 5.19 | 0.87 |
| Abdominal compartment syndrome | Gastrointestinal disorders | 28.35 | 4 | 4.81 | 0.83 |
| Slow response to stimuli | Nervous system disorders | 13.33 | 4 | 3.73 | 0.65 |
| Anisocoria | Eye disorders | 11.41 | 4 | 3.51 | 0.59 |
| Vasoplegia syndrome | Injury, poisoning and procedural complications | 11.21 | 4 | 3.48 | 0.59 |
| Quadriparesis | Nervous system disorders | 10.39 | 4 | 3.37 | 0.56 |
| Clonus | Nervous system disorders | 6.18 | 4 | 2.63 | 0.31 |
| Mucormycosis | Infections and infestations | 5.83 | 4 | 2.54 | 0.28 |
| Pneumonia viral | Infections and infestations | 5.55 | 4 | 2.47 | 0.25 |
| Azotaemia | Renal and urinary disorders | 4.36 | 4 | 2.12 | 0.09 |
| Cytomegalovirus viraemia | Infections and infestations | 4.24 | 4 | 2.08 | 0.07 |
| Immune effector cell-associated hlh-like syndrome | Immune system disorders | 242.58 | 3 | 7.83 | 0.48 |
| Fusobacterium infection | Infections and infestations | 108.91 | 3 | 6.72 | 0.49 |
| Bone marrow infiltration | Blood and lymphatic system disorders | 35.46 | 3 | 5.13 | 0.43 |
| Bacillus infection | Infections and infestations | 21.05 | 3 | 4.39 | 0.36 |
| Serratia infection | Infections and infestations | 17.64 | 3 | 4.13 | 0.32 |
| Cranial nerve disorder | Nervous system disorders | 17.41 | 3 | 4.12 | 0.32 |
| Abdominal lymphadenopathy | Blood and lymphatic system disorders | 15.03 | 3 | 3.9 | 0.29 |
| Klebsiella bacteraemia | Infections and infestations | 14.76 | 3 | 3.88 | 0.28 |
| Haemophilus infection | Infections and infestations | 13.61 | 3 | 3.76 | 0.26 |
| Gastrointestinal viral infection | Infections and infestations | 13 | 3 | 3.7 | 0.25 |
| Gastrointestinal bacterial infection | Infections and infestations | 11.81 | 3 | 3.56 | 0.23 |
| Moaning | General disorders and administration site conditions | 11.44 | 3 | 3.51 | 0.22 |
| Encephalitis viral | Infections and infestations | 11.2 | 3 | 3.48 | 0.21 |
| Sialoadenitis | Infections and infestations | 9.76 | 3 | 3.28 | 0.17 |
| Apraxia | Nervous system disorders | 8.71 | 3 | 3.12 | 0.13 |
| Fungaemia | Infections and infestations | 8.5 | 3 | 3.08 | 0.12 |
| Pupil fixed | Eye disorders | 7.59 | 3 | 2.92 | 0.07 |
| Pseudomonal sepsis | Infections and infestations | 7.54 | 3 | 2.91 | 0.07 |
| Pupillary reflex impaired | Eye disorders | 7.3 | 3 | 2.86 | 0.06 |
| Distributive shock | Vascular disorders | 6.69 | 3 | 2.74 | 0.02 |
| Varicella zoster virus infection | Infections and infestations | 6.59 | 3 | 2.72 | 0.01 |
| Hyperphosphataemia | Metabolism and nutrition disorders | 6.5 | 3 | 2.7 | 0.01 |

**Table S3** PT and SOC for the positive signal detection of Tecartus in the FAERS database

| PT | SOC | PRR | n | IC | IC025 |
| --- | --- | --- | --- | --- | --- |
| Cytokine release syndrome | Immune system disorders | 306.78 | 641 | 8.18 | 7.51 |
| Immune effector cell-associated neurotoxicity syndrome | Nervous system disorders | 664.74 | 439 | 9.2 | 7.82 |
| Neurotoxicity | Nervous system disorders | 120.08 | 163 | 6.87 | 5.87 |
| Death | General disorders and administration site conditions | 2.03 | 119 | 1.02 | 0.74 |
| Pyrexia | General disorders and administration site conditions | 4.05 | 86 | 2.02 | 1.65 |
| Hypotension | Vascular disorders | 6.71 | 81 | 2.75 | 2.33 |
| Confusional state | Psychiatric disorders | 5.71 | 51 | 2.51 | 1.98 |
| Encephalopathy | Nervous system disorders | 30.69 | 48 | 4.93 | 3.83 |
| Hypoxia | Respiratory, thoracic and mediastinal disorders | 17.52 | 41 | 4.13 | 3.2 |
| Pancytopenia | Blood and lymphatic system disorders | 11.44 | 39 | 3.51 | 2.72 |
| Tremor | Nervous system disorders | 4.5 | 39 | 2.17 | 1.59 |
| Tachycardia | Cardiac disorders | 7.28 | 38 | 2.86 | 2.18 |
| Acute kidney injury | Renal and urinary disorders | 2.96 | 37 | 1.56 | 1.02 |
| Aphasia | Nervous system disorders | 19.47 | 32 | 4.28 | 3.13 |
| Neutropenia | Blood and lymphatic system disorders | 2.65 | 29 | 1.4 | 0.8 |
| Seizure | Nervous system disorders | 2.94 | 27 | 1.55 | 0.91 |
| Infection | Infections and infestations | 2.66 | 27 | 1.41 | 0.78 |
| Haemophagocytic lymphohistiocytosis | Immune system disorders | 29.41 | 26 | 4.87 | 3.28 |
| Febrile neutropenia | Blood and lymphatic system disorders | 5.1 | 25 | 2.35 | 1.57 |
| Sepsis | Infections and infestations | 3.84 | 25 | 1.94 | 1.22 |
| Infusion related reaction | Injury, poisoning and procedural complications | 3.64 | 21 | 1.86 | 1.08 |
| Brain oedema | Nervous system disorders | 28.59 | 20 | 4.83 | 2.99 |
| Mental status changes | Psychiatric disorders | 15.06 | 20 | 3.91 | 2.54 |
| Depressed level of consciousness | Nervous system disorders | 8.84 | 20 | 3.14 | 2.05 |
| Thrombocytopenia | Blood and lymphatic system disorders | 2.85 | 20 | 1.51 | 0.76 |
| Multiple organ dysfunction syndrome | General disorders and administration site conditions | 7.6 | 19 | 2.93 | 1.87 |
| Agitation | Psychiatric disorders | 5.73 | 19 | 2.52 | 1.56 |
| Respiratory failure | Respiratory, thoracic and mediastinal disorders | 4.51 | 18 | 2.17 | 1.26 |
| Delirium | Psychiatric disorders | 7.83 | 17 | 2.97 | 1.82 |
| Cytopenia | Blood and lymphatic system disorders | 13.45 | 16 | 3.75 | 2.25 |
| Septic shock | Infections and infestations | 5.33 | 15 | 2.41 | 1.34 |
| Pleural effusion | Respiratory, thoracic and mediastinal disorders | 4.53 | 15 | 2.18 | 1.17 |
| Hypofibrinogenaemia | Blood and lymphatic system disorders | 192.78 | 14 | 7.54 | 3.04 |
| Atrial fibrillation | Cardiac disorders | 2.3 | 14 | 1.2 | 0.34 |
| Tachypnoea | Respiratory, thoracic and mediastinal disorders | 13.01 | 12 | 3.7 | 1.95 |
| Unresponsive to stimuli | Nervous system disorders | 8.69 | 12 | 3.12 | 1.65 |
| Hyperbilirubinaemia | Hepatobiliary disorders | 17.94 | 11 | 4.16 | 2.06 |
| Disorientation | Psychiatric disorders | 6.08 | 11 | 2.6 | 1.26 |
| Lethargy | Nervous system disorders | 3.79 | 11 | 1.92 | 0.78 |
| Hyponatraemia | Metabolism and nutrition disorders | 3.5 | 11 | 1.81 | 0.7 |
| Bacteraemia | Infections and infestations | 12.02 | 10 | 3.58 | 1.71 |
| Shock | Vascular disorders | 7.64 | 10 | 2.93 | 1.38 |
| Bradycardia | Cardiac disorders | 3.27 | 10 | 1.71 | 0.56 |
| Cytomegalovirus infection reactivation | Infections and infestations | 14.78 | 9 | 3.88 | 1.72 |
| Nervous system disorder | Nervous system disorders | 7.86 | 9 | 2.97 | 1.3 |
| Dysgraphia | Nervous system disorders | 19.87 | 8 | 4.31 | 1.72 |
| Physical deconditioning | General disorders and administration site conditions | 21.75 | 7 | 4.44 | 1.57 |
| Incontinence | Renal and urinary disorders | 11.88 | 7 | 3.57 | 1.31 |
| Bone marrow failure | Blood and lymphatic system disorders | 11.56 | 7 | 3.53 | 1.29 |
| Tumour lysis syndrome | Metabolism and nutrition disorders | 10.84 | 7 | 3.44 | 1.26 |
| Acute respiratory failure | Respiratory, thoracic and mediastinal disorders | 5.1 | 7 | 2.35 | 0.73 |
| Mucormycosis | Infections and infestations | 29.37 | 6 | 4.87 | 1.44 |
| Disseminated intravascular coagulation | Blood and lymphatic system disorders | 9.79 | 6 | 3.29 | 1.02 |
| Hypervolaemia | Metabolism and nutrition disorders | 6.97 | 6 | 2.8 | 0.82 |
| Hyperglycaemia | Metabolism and nutrition disorders | 3.07 | 6 | 1.62 | 0.15 |
| Covid-19 pneumonia | Infections and infestations | 3.03 | 6 | 1.6 | 0.14 |
| Cytokine storm | Immune system disorders | 68.85 | 5 | 6.09 | 1.29 |
| Cytomegalovirus viraemia | Infections and infestations | 13.15 | 5 | 3.71 | 0.94 |
| Acidosis | Metabolism and nutrition disorders | 12.01 | 5 | 3.58 | 0.9 |
| Hypoalbuminaemia | Metabolism and nutrition disorders | 10.72 | 5 | 3.42 | 0.85 |
| Hypogammaglobulinaemia | Immune system disorders | 9 | 5 | 3.17 | 0.77 |
| Status epilepticus | Nervous system disorders | 7.48 | 5 | 2.9 | 0.67 |
| Hemiparesis | Nervous system disorders | 6.31 | 5 | 2.66 | 0.56 |
| Immune effector cell-associated haematotoxicity | Blood and lymphatic system disorders | 555.38 | 4 | 8.97 | 0.94 |
| Fungaemia | Infections and infestations | 36.62 | 4 | 5.18 | 0.87 |
| Hyperuricaemia | Metabolism and nutrition disorders | 16.24 | 4 | 4.02 | 0.71 |
| Hypernatraemia | Metabolism and nutrition disorders | 13.17 | 4 | 3.72 | 0.64 |
| Atelectasis | Respiratory, thoracic and mediastinal disorders | 8.93 | 4 | 3.16 | 0.49 |
| Vascular device infection | Infections and infestations | 6.92 | 4 | 2.79 | 0.37 |
| Bronchopulmonary aspergillosis | Infections and infestations | 6.75 | 4 | 2.75 | 0.36 |
| Facial paralysis | Nervous system disorders | 5.78 | 4 | 2.53 | 0.27 |
| Ventricular tachycardia | Cardiac disorders | 4.47 | 4 | 2.16 | 0.11 |
| Ischaemic stroke | Nervous system disorders | 4.37 | 4 | 2.13 | 0.09 |
| Cell-mediated cytotoxicity | Metabolism and nutrition disorders | 999.68 | 3 | 9.7 | 0.41 |
| Immune effector cell-associated hlh-like syndrome | Immune system disorders | 340.8 | 3 | 8.32 | 0.48 |
| Diverticulitis intestinal perforated | Infections and infestations | 64.08 | 3 | 5.98 | 0.48 |
| Human herpesvirus 6 infection | Infections and infestations | 28.62 | 3 | 4.83 | 0.41 |
| Azotaemia | Renal and urinary disorders | 14.29 | 3 | 3.83 | 0.28 |
| Staphylococcal bacteraemia | Infections and infestations | 10.07 | 3 | 3.33 | 0.18 |
| Apnoea | Respiratory, thoracic and mediastinal disorders | 8.48 | 3 | 3.08 | 0.12 |
| Neutropenic sepsis | Infections and infestations | 7.21 | 3 | 2.85 | 0.05 |

**Table S4** PT and SOC for the positive signal detection of Yescarta in the FAERS database

| PT | SOC | PRR | n | IC | IC025 |
| --- | --- | --- | --- | --- | --- |
| Cytokine release syndrome | Immune system disorders | 834.82 | 2474 | 9.4 | 8.99 |
| Immune effector cell-associated neurotoxicity syndrome | Nervous system disorders | 1624.36 | 1207 | 10.12 | 9.08 |
| Neurotoxicity | Nervous system disorders | 242.99 | 875 | 7.83 | 7.39 |
| Death | General disorders and administration site conditions | 2.56 | 514 | 1.35 | 1.22 |
| Pyrexia | General disorders and administration site conditions | 5.23 | 421 | 2.39 | 2.23 |
| Hypotension | Vascular disorders | 4.41 | 206 | 2.14 | 1.92 |
| Neutropenia | Blood and lymphatic system disorders | 6.11 | 191 | 2.61 | 2.36 |
| Encephalopathy | Nervous system disorders | 30.67 | 174 | 4.93 | 4.48 |
| Confusional state | Psychiatric disorders | 4.31 | 164 | 2.11 | 1.85 |
| Pancytopenia | Blood and lymphatic system disorders | 11.68 | 150 | 3.54 | 3.21 |
| Aphasia | Nervous system disorders | 17.95 | 130 | 4.16 | 3.73 |
| Tremor | Nervous system disorders | 3.21 | 126 | 1.68 | 1.4 |
| Thrombocytopenia | Blood and lymphatic system disorders | 4.04 | 104 | 2.01 | 1.69 |
| Hypoxia | Respiratory, thoracic and mediastinal disorders | 12.32 | 99 | 3.62 | 3.17 |
| Tachycardia | Cardiac disorders | 4.56 | 94 | 2.19 | 1.84 |
| Cytopenia | Blood and lymphatic system disorders | 36.94 | 93 | 5.19 | 4.43 |
| Febrile neutropenia | Blood and lymphatic system disorders | 5.57 | 85 | 2.47 | 2.09 |
| Infection | Infections and infestations | 2.56 | 84 | 1.36 | 1.02 |
| Haemophagocytic lymphohistiocytosis | Immune system disorders | 33.55 | 75 | 5.06 | 4.21 |
| Sepsis | Infections and infestations | 2.75 | 72 | 1.46 | 1.09 |
| Septic shock | Infections and infestations | 6.93 | 69 | 2.79 | 2.33 |
| Disorientation | Psychiatric disorders | 6.04 | 57 | 2.59 | 2.09 |
| Atrial fibrillation | Cardiac disorders | 2.49 | 57 | 1.32 | 0.9 |
| Multiple organ dysfunction syndrome | General disorders and administration site conditions | 4.94 | 52 | 2.3 | 1.8 |
| Mental status changes | Psychiatric disorders | 7.78 | 51 | 2.96 | 2.38 |
| Hypogammaglobulinaemia | Immune system disorders | 36.76 | 49 | 5.19 | 4 |
| Pleural effusion | Respiratory, thoracic and mediastinal disorders | 3.4 | 49 | 1.76 | 1.29 |
| Agitation | Psychiatric disorders | 2.73 | 48 | 1.45 | 0.99 |
| Dysgraphia | Nervous system disorders | 28.17 | 44 | 4.8 | 3.7 |
| Respiratory failure | Respiratory, thoracic and mediastinal disorders | 2.49 | 43 | 1.32 | 0.83 |
| Bone marrow failure | Blood and lymphatic system disorders | 7.83 | 39 | 2.97 | 2.28 |
| Infusion related reaction | Injury, poisoning and procedural complications | 2.55 | 38 | 1.35 | 0.83 |
| Brain oedema | Nervous system disorders | 12.7 | 37 | 3.66 | 2.81 |
| Depressed level of consciousness | Nervous system disorders | 4.12 | 37 | 2.04 | 1.46 |
| Nervous system disorder | Nervous system disorders | 4.69 | 36 | 2.23 | 1.62 |
| Bacteraemia | Infections and infestations | 11.6 | 31 | 3.53 | 2.61 |
| Cytokine storm | Immune system disorders | 204.42 | 30 | 7.6 | 4.21 |
| Hypoalbuminaemia | Metabolism and nutrition disorders | 17.24 | 30 | 4.1 | 2.98 |
| Delirium | Psychiatric disorders | 3.5 | 28 | 1.8 | 1.15 |
| Hyponatraemia | Metabolism and nutrition disorders | 2.13 | 28 | 1.09 | 0.5 |
| Cardiorenal syndrome | Cardiac disorders | 216.87 | 27 | 7.68 | 4.07 |
| Incontinence | Renal and urinary disorders | 10.97 | 27 | 3.45 | 2.47 |
| Disseminated intravascular coagulation | Blood and lymphatic system disorders | 7.79 | 26 | 2.96 | 2.08 |
| Hyperbilirubinaemia | Hepatobiliary disorders | 9.31 | 22 | 3.22 | 2.17 |
| Unresponsive to stimuli | Nervous system disorders | 3.54 | 22 | 1.82 | 1.07 |
| Tachypnoea | Respiratory, thoracic and mediastinal disorders | 6.46 | 20 | 2.69 | 1.73 |
| Agranulocytosis | Blood and lymphatic system disorders | 4.82 | 20 | 2.27 | 1.4 |
| Hypervolaemia | Metabolism and nutrition disorders | 4.33 | 20 | 2.11 | 1.27 |
| Coagulopathy | Blood and lymphatic system disorders | 4.86 | 19 | 2.28 | 1.38 |
| Acute respiratory failure | Respiratory, thoracic and mediastinal disorders | 4.32 | 19 | 2.11 | 1.24 |
| Cytomegalovirus infection reactivation | Infections and infestations | 19.89 | 17 | 4.31 | 2.59 |
| Cytomegalovirus viraemia | Infections and infestations | 18.05 | 17 | 4.17 | 2.53 |
| Shock | Vascular disorders | 3.28 | 17 | 1.71 | 0.86 |
| Hypofibrinogenaemia | Blood and lymphatic system disorders | 71.72 | 16 | 6.14 | 3.08 |
| Orthostatic hypotension | Vascular disorders | 3.91 | 16 | 1.97 | 1.04 |
| Clostridium difficile infection | Infections and infestations | 3.27 | 16 | 1.71 | 0.83 |
| Clostridium difficile colitis | Infections and infestations | 5.97 | 15 | 2.58 | 1.46 |
| Status epilepticus | Nervous system disorders | 5.75 | 15 | 2.52 | 1.43 |
| Neurological symptom | Nervous system disorders | 10.71 | 14 | 3.42 | 1.95 |
| Aspergillus infection | Infections and infestations | 8 | 14 | 3 | 1.7 |
| Pulseless electrical activity | Cardiac disorders | 11.3 | 13 | 3.49 | 1.93 |
| Ventricular tachycardia | Cardiac disorders | 3.34 | 13 | 1.74 | 0.74 |
| Hemiparesis | Nervous system disorders | 3.24 | 13 | 1.69 | 0.71 |
| Human herpesvirus 6 encephalitis | Infections and infestations | 202.87 | 12 | 7.58 | 2.79 |
| Physical deconditioning | General disorders and administration site conditions | 23.36 | 12 | 4.54 | 2.3 |
| Bradyphrenia | Psychiatric disorders | 7.6 | 12 | 2.92 | 1.53 |
| Cytomegalovirus infection | Infections and infestations | 3.14 | 12 | 1.65 | 0.63 |
| Fungaemia | Infections and infestations | 31.29 | 11 | 4.96 | 2.31 |
| Aspiration | Respiratory, thoracic and mediastinal disorders | 4.47 | 11 | 2.16 | 0.96 |
| Intestinal perforation | Gastrointestinal disorders | 4.17 | 11 | 2.06 | 0.89 |
| Acute respiratory distress syndrome | Respiratory, thoracic and mediastinal disorders | 2.65 | 11 | 1.41 | 0.39 |
| Immune effector cell-associated haematotoxicity | Blood and lymphatic system disorders | 1183.39 | 10 | 9.79 | 2.46 |
| Systemic candida | Infections and infestations | 18.42 | 10 | 4.2 | 1.96 |
| Enterococcal infection | Infections and infestations | 8.95 | 10 | 3.16 | 1.5 |
| Tumour lysis syndrome | Metabolism and nutrition disorders | 5.15 | 10 | 2.36 | 1.03 |
| Progressive multifocal leukoencephalopathy | Infections and infestations | 5.15 | 10 | 2.36 | 1.03 |
| Adrenal insufficiency | Endocrine disorders | 3.84 | 10 | 1.94 | 0.74 |
| Bacterial infection | Infections and infestations | 2.37 | 10 | 1.25 | 0.21 |
| Candida infection | Infections and infestations | 2.11 | 10 | 1.08 | 0.07 |
| Systemic mycosis | Infections and infestations | 34.81 | 9 | 5.11 | 2.07 |
| Escherichia bacteraemia | Infections and infestations | 17.15 | 9 | 4.09 | 1.8 |
| Pneumonia fungal | Infections and infestations | 8.59 | 9 | 3.1 | 1.37 |
| Supraventricular tachycardia | Cardiac disorders | 4 | 9 | 2 | 0.71 |
| Bacterial sepsis | Infections and infestations | 10.5 | 8 | 3.39 | 1.39 |
| Febrile bone marrow aplasia | Blood and lymphatic system disorders | 8.43 | 8 | 3.07 | 1.24 |
| Bronchopulmonary aspergillosis | Infections and infestations | 4.51 | 8 | 2.17 | 0.73 |
| Escherichia infection | Infections and infestations | 4.36 | 8 | 2.12 | 0.7 |
| Cardiomyopathy | Cardiac disorders | 2.25 | 8 | 1.17 | 0.02 |
| Paraplegia | Nervous system disorders | 10.22 | 7 | 3.35 | 1.22 |
| Staphylococcal bacteraemia | Infections and infestations | 7.23 | 7 | 2.85 | 1 |
| Vascular device infection | Infections and infestations | 6.83 | 7 | 2.77 | 0.96 |
| Hypertransaminasaemia | Hepatobiliary disorders | 5 | 7 | 2.32 | 0.71 |
| Shock haemorrhagic | Vascular disorders | 3.77 | 7 | 1.91 | 0.46 |
| Ileus | Gastrointestinal disorders | 2.51 | 7 | 1.33 | 0.06 |
| Immune effector cell-associated hlh-like syndrome | Immune system disorders | 519.54 | 6 | 8.82 | 1.62 |
| Pseudomonal bacteraemia | Infections and infestations | 32.32 | 6 | 5 | 1.46 |
| Congenital aplasia | Congenital, familial and genetic disorders | 10.99 | 6 | 3.45 | 1.08 |
| Metabolic encephalopathy | Nervous system disorders | 8.72 | 6 | 3.12 | 0.96 |
| Brain herniation | Injury, poisoning and procedural complications | 8.61 | 6 | 3.1 | 0.95 |
| Hyperpyrexia | General disorders and administration site conditions | 6.69 | 6 | 2.74 | 0.79 |
| Klebsiella infection | Infections and infestations | 5.3 | 6 | 2.41 | 0.62 |
| Stress cardiomyopathy | Cardiac disorders | 4.49 | 6 | 2.16 | 0.49 |
| Haemodynamic instability | Vascular disorders | 3.55 | 6 | 1.83 | 0.29 |
| Acidosis | Metabolism and nutrition disorders | 3.35 | 6 | 1.74 | 0.23 |
| Pulmonary haemorrhage | Respiratory, thoracic and mediastinal disorders | 3.2 | 6 | 1.68 | 0.19 |
| Sensory loss | Nervous system disorders | 3.18 | 6 | 1.67 | 0.18 |
| Pneumonia bacterial | Infections and infestations | 2.93 | 6 | 1.55 | 0.11 |
| Lung infiltration | Respiratory, thoracic and mediastinal disorders | 2.83 | 6 | 1.5 | 0.07 |
| Cerebellar infarction | Nervous system disorders | 12.25 | 5 | 3.61 | 0.91 |
| Human herpesvirus 6 infection | Infections and infestations | 11.57 | 5 | 3.53 | 0.88 |
| Adenovirus infection | Infections and infestations | 7.95 | 5 | 2.99 | 0.7 |
| Bk virus infection | Infections and infestations | 6.58 | 5 | 2.72 | 0.59 |
| Failure to thrive | Metabolism and nutrition disorders | 3.9 | 5 | 1.96 | 0.22 |
| Intestinal ischaemia | Gastrointestinal disorders | 3.9 | 5 | 1.96 | 0.21 |
| Intracranial pressure increased | Nervous system disorders | 3.88 | 5 | 1.96 | 0.21 |
| Adenoviral haemorrhagic cystitis | Infections and infestations | 83.04 | 4 | 6.34 | 0.94 |
| Enterococcal sepsis | Infections and infestations | 20.43 | 4 | 4.34 | 0.77 |
| Urinary tract infection enterococcal | Infections and infestations | 17.64 | 4 | 4.13 | 0.73 |
| Enterococcal bacteraemia | Infections and infestations | 16.57 | 4 | 4.04 | 0.71 |
| Apraxia | Nervous system disorders | 11.6 | 4 | 3.53 | 0.6 |
| Myelitis | Infections and infestations | 11.49 | 4 | 3.52 | 0.6 |
| Parainfluenzae virus infection | Infections and infestations | 10.95 | 4 | 3.45 | 0.58 |
| Hypoproteinaemia | Metabolism and nutrition disorders | 9.49 | 4 | 3.24 | 0.52 |
| Pneumoperitoneum | Gastrointestinal disorders | 9.46 | 4 | 3.24 | 0.52 |
| Pneumonia pseudomonal | Infections and infestations | 8.47 | 4 | 3.08 | 0.47 |
| Myelopathy | Nervous system disorders | 7.9 | 4 | 2.98 | 0.44 |
| Retroperitoneal haematoma | Gastrointestinal disorders | 7.7 | 4 | 2.94 | 0.42 |
| Device related sepsis | Infections and infestations | 7.43 | 4 | 2.89 | 0.41 |
| Abdominal mass | Gastrointestinal disorders | 6.72 | 4 | 2.75 | 0.35 |
| Mucormycosis | Infections and infestations | 5.82 | 4 | 2.54 | 0.27 |
| Bone marrow disorder | Blood and lymphatic system disorders | 5.68 | 4 | 2.5 | 0.26 |
| Cardiac dysfunction | Cardiac disorders | 5.21 | 4 | 2.38 | 0.21 |
| Leukoencephalopathy | Nervous system disorders | 4.52 | 4 | 2.17 | 0.11 |
| Cystitis haemorrhagic | Renal and urinary disorders | 4.19 | 4 | 2.07 | 0.06 |
| Cerebral atrophy | Nervous system disorders | 4.14 | 4 | 2.05 | 0.05 |
| Apheresis related complication | Injury, poisoning and procedural complications | 968.23 | 3 | 9.57 | 0.37 |
| Acalculia | Nervous system disorders | 242.06 | 3 | 7.82 | 0.48 |
| Graft versus host disease in liver | Immune system disorders | 18.65 | 3 | 4.21 | 0.34 |
| Haemophilus infection | Infections and infestations | 13.58 | 3 | 3.76 | 0.26 |
| Encephalitis herpes | Infections and infestations | 13.58 | 3 | 3.76 | 0.26 |
| Brain stem infarction | Nervous system disorders | 11.74 | 3 | 3.55 | 0.22 |
| Klebsiella sepsis | Infections and infestations | 11.38 | 3 | 3.5 | 0.22 |
| Hypertensive emergency | Vascular disorders | 11.18 | 3 | 3.48 | 0.21 |
| Central nervous system infection | Infections and infestations | 11.18 | 3 | 3.48 | 0.21 |
| Myelitis transverse | Nervous system disorders | 9.66 | 3 | 3.27 | 0.16 |
| Anisocoria | Eye disorders | 8.53 | 3 | 3.09 | 0.12 |
| Pupil fixed | Eye disorders | 7.57 | 3 | 2.92 | 0.07 |
| Haematological infection | Infections and infestations | 7.28 | 3 | 2.86 | 0.06 |
| Small intestinal perforation | Gastrointestinal disorders | 6.89 | 3 | 2.78 | 0.03 |

**Table S5** PT and SOC for the positive signal detection of Abecma in the FAERS database

| PT | SOC | PRR | n | IC | IC025 |
| --- | --- | --- | --- | --- | --- |
| Cytokine release syndrome | Immune system disorders | 828.35 | 494 | 9.64 | 8.11 |
| Fatigue | General disorders and administration site conditions | 4.97 | 151 | 2.31 | 2.03 |
| Immune effector cell-associated neurotoxicity syndrome | Nervous system disorders | 833.26 | 147 | 9.65 | 6.72 |
| Pyrexia | General disorders and administration site conditions | 3.96 | 54 | 1.98 | 1.51 |
| Headache | Nervous system disorders | 2.11 | 52 | 1.08 | 0.65 |
| Decreased appetite | Metabolism and nutrition disorders | 5.22 | 50 | 2.38 | 1.86 |
| Neurotoxicity | Nervous system disorders | 73.92 | 48 | 6.2 | 4.47 |
| Neutropenia | Blood and lymphatic system disorders | 8.3 | 44 | 3.05 | 2.4 |
| Anaemia | Blood and lymphatic system disorders | 5.4 | 41 | 2.43 | 1.84 |
| Hypogammaglobulinaemia | Immune system disorders | 127.92 | 29 | 6.99 | 4.08 |
| Acute kidney injury | Renal and urinary disorders | 3.62 | 28 | 1.86 | 1.19 |
| Hypotension | Vascular disorders | 3.54 | 28 | 1.82 | 1.16 |
| Tremor | Nervous system disorders | 4.06 | 27 | 2.02 | 1.32 |
| Tachycardia | Cardiac disorders | 6.87 | 24 | 2.78 | 1.89 |
| Haemophagocytic lymphohistiocytosis | Immune system disorders | 55.09 | 21 | 5.78 | 3.37 |
| Somnolence | Nervous system disorders | 2.64 | 21 | 1.4 | 0.68 |
| Myalgia | Musculoskeletal and connective tissue disorders | 2.96 | 20 | 1.57 | 0.81 |
| Hypoalbuminaemia | Metabolism and nutrition disorders | 64.34 | 19 | 6 | 3.3 |
| Cytopenia | Blood and lymphatic system disorders | 44.19 | 19 | 5.46 | 3.16 |
| Pancytopenia | Blood and lymphatic system disorders | 8.71 | 19 | 3.12 | 2 |
| Chills | General disorders and administration site conditions | 3.86 | 18 | 1.95 | 1.08 |
| Infusion related reaction | Injury, poisoning and procedural complications | 5.95 | 15 | 2.57 | 1.46 |
| Thrombocytopenia | Blood and lymphatic system disorders | 3.21 | 14 | 1.68 | 0.74 |
| Confusional state | Psychiatric disorders | 2.17 | 14 | 1.12 | 0.26 |
| Haematotoxicity | Blood and lymphatic system disorders | 38.58 | 13 | 5.27 | 2.61 |
| Febrile bone marrow aplasia | Blood and lymphatic system disorders | 74.78 | 12 | 6.22 | 2.68 |
| Sepsis | Infections and infestations | 2.71 | 12 | 1.44 | 0.46 |
| Immunodeficiency | Immune system disorders | 18.2 | 10 | 4.18 | 1.95 |
| Hypoxia | Respiratory, thoracic and mediastinal disorders | 7.33 | 10 | 2.87 | 1.34 |
| Febrile neutropenia | Blood and lymphatic system disorders | 3.48 | 9 | 1.8 | 0.56 |
| Agitation | Psychiatric disorders | 3.02 | 9 | 1.6 | 0.42 |
| Balance disorder | Nervous system disorders | 2.61 | 9 | 1.39 | 0.26 |
| Altered state of consciousness | Nervous system disorders | 9.42 | 8 | 3.24 | 1.32 |
| Encephalopathy | Nervous system disorders | 8.26 | 8 | 3.05 | 1.23 |
| Laryngeal oedema | Respiratory, thoracic and mediastinal disorders | 29.32 | 7 | 4.87 | 1.67 |
| Parkinsonism | Nervous system disorders | 18.11 | 7 | 4.18 | 1.51 |
| Disseminated intravascular coagulation | Blood and lymphatic system disorders | 12.36 | 7 | 3.63 | 1.33 |
| Pleural effusion | Respiratory, thoracic and mediastinal disorders | 2.87 | 7 | 1.52 | 0.19 |
| Hyperbilirubinaemia | Hepatobiliary disorders | 14.97 | 6 | 3.9 | 1.23 |
| Aphasia | Nervous system disorders | 4.87 | 6 | 2.28 | 0.56 |
| Arrhythmia | Cardiac disorders | 3.12 | 6 | 1.64 | 0.17 |
| Cytomegalovirus chorioretinitis | Infections and infestations | 68.82 | 5 | 6.1 | 1.3 |
| Malnutrition | Metabolism and nutrition disorders | 11.58 | 5 | 3.53 | 0.89 |
| Tachypnoea | Respiratory, thoracic and mediastinal disorders | 9.53 | 5 | 3.25 | 0.8 |
| Myelosuppression | Blood and lymphatic system disorders | 4.5 | 5 | 2.17 | 0.33 |
| Rhinovirus infection | Infections and infestations | 30.25 | 4 | 4.92 | 0.85 |
| Cytomegalovirus viraemia | Infections and infestations | 24.98 | 4 | 4.64 | 0.81 |
| Viral upper respiratory tract infection | Infections and infestations | 19.43 | 4 | 4.28 | 0.76 |
| Incontinence | Renal and urinary disorders | 9.57 | 4 | 3.26 | 0.53 |
| Hypervolaemia | Metabolism and nutrition disorders | 5.11 | 4 | 2.35 | 0.2 |
| Bone marrow failure | Blood and lymphatic system disorders | 4.73 | 4 | 2.24 | 0.15 |
| Hepatotoxicity | Hepatobiliary disorders | 4.69 | 4 | 2.23 | 0.14 |
| Escherichia bacteraemia | Infections and infestations | 33.65 | 3 | 5.07 | 0.43 |
| Cytomegalovirus infection reactivation | Infections and infestations | 20.63 | 3 | 4.36 | 0.36 |
| Tumour lysis syndrome | Metabolism and nutrition disorders | 9.12 | 3 | 3.19 | 0.14 |
| Clostridium difficile colitis | Infections and infestations | 7.05 | 3 | 2.82 | 0.04 |
| Bacteraemia | Infections and infestations | 6.61 | 3 | 2.72 | 0.02 |

**Table S6** PT and SOC for the positive signal detection of Carvykti in the FAERS database

| PT | SOC | PRR | n | IC | IC025 |
| --- | --- | --- | --- | --- | --- |
| Cytokine release syndrome | Immune system disorders | 311.88 | 423 | 8.24 | 7.32 |
| Pyrexia | General disorders and administration site conditions | 5.8 | 179 | 2.54 | 2.28 |
| Immune effector cell-associated neurotoxicity syndrome | Nervous system disorders | 407.73 | 162 | 8.61 | 6.61 |
| Parkinsonism | Nervous system disorders | 177.1 | 152 | 7.44 | 6.11 |
| Neurotoxicity | Nervous system disorders | 71.12 | 104 | 6.14 | 5.12 |
| Malaise | General disorders and administration site conditions | 2.36 | 94 | 1.24 | 0.92 |
| Cranial nerve paralysis | Nervous system disorders | 3826.87 | 90 | 11.4 | 6.11 |
| Facial paralysis | Nervous system disorders | 58.2 | 74 | 5.85 | 4.7 |
| Bell's palsy | Nervous system disorders | 571.77 | 66 | 9.07 | 5.53 |
| Haemophagocytic lymphohistiocytosis | Immune system disorders | 74.65 | 64 | 6.21 | 4.76 |
| Covid-19 | Infections and infestations | 3.79 | 58 | 1.92 | 1.48 |
| Illness | General disorders and administration site conditions | 7.26 | 57 | 2.86 | 2.33 |
| Infection | Infections and infestations | 4.14 | 52 | 2.05 | 1.57 |
| Infusion related reaction | Injury, poisoning and procedural complications | 7.72 | 44 | 2.95 | 2.31 |
| Cytopenia | Blood and lymphatic system disorders | 44.35 | 43 | 5.46 | 4.04 |
| Colitis | Gastrointestinal disorders | 12.75 | 42 | 3.67 | 2.88 |
| Neutropenia | Blood and lymphatic system disorders | 3.17 | 38 | 1.66 | 1.12 |
| Hypotension | Vascular disorders | 2.01 | 36 | 1.01 | 0.49 |
| Sepsis | Infections and infestations | 3.49 | 35 | 1.8 | 1.22 |
| Thrombocytopenia | Blood and lymphatic system disorders | 3.04 | 30 | 1.61 | 0.99 |
| Immune-mediated enterocolitis | Gastrointestinal disorders | 105.51 | 27 | 6.71 | 3.93 |
| Respiratory failure | Respiratory, thoracic and mediastinal disorders | 4.09 | 27 | 2.03 | 1.33 |
| Bacterial infection | Infections and infestations | 16.13 | 26 | 4.01 | 2.81 |
| Febrile neutropenia | Blood and lymphatic system disorders | 3.93 | 23 | 1.97 | 1.22 |
| Pancytopenia | Blood and lymphatic system disorders | 4.46 | 22 | 2.16 | 1.35 |
| Unevaluable event | General disorders and administration site conditions | 2.99 | 21 | 1.58 | 0.84 |
| Guillain-barre syndrome | Nervous system disorders | 48.23 | 20 | 5.58 | 3.26 |
| Influenza | Infections and infestations | 2.06 | 20 | 1.05 | 0.34 |
| Immune effector cell-associated hlh-like syndrome | Immune system disorders | 5753.51 | 18 | 11.79 | 3.46 |
| Septic shock | Infections and infestations | 4.45 | 17 | 2.15 | 1.22 |
| Viral infection | Infections and infestations | 5.53 | 16 | 2.47 | 1.43 |
| Brain fog | Nervous system disorders | 16.1 | 15 | 4.01 | 2.33 |
| Encephalopathy | Nervous system disorders | 6.39 | 14 | 2.68 | 1.49 |
| Nervous system disorder | Nervous system disorders | 4.42 | 13 | 2.14 | 1.06 |
| Fungal infection | Infections and infestations | 4.4 | 13 | 2.14 | 1.05 |
| Hypoxia | Respiratory, thoracic and mediastinal disorders | 4.21 | 13 | 2.07 | 1 |
| Acute respiratory failure | Respiratory, thoracic and mediastinal disorders | 7.11 | 12 | 2.83 | 1.47 |
| Diplopia | Eye disorders | 5.23 | 12 | 2.39 | 1.18 |
| Clostridium difficile infection | Infections and infestations | 5.87 | 11 | 2.55 | 1.23 |
| Facial nerve disorder | Nervous system disorders | 258.93 | 10 | 7.98 | 2.52 |
| Hypogammaglobulinaemia | Immune system disorders | 19.43 | 10 | 4.28 | 1.99 |
| Mental status changes | Psychiatric disorders | 3.97 | 10 | 1.99 | 0.77 |
| Multiple organ dysfunction syndrome | General disorders and administration site conditions | 2.48 | 10 | 1.31 | 0.26 |
| Speech disorder | Nervous system disorders | 2.11 | 10 | 1.08 | 0.07 |
| Lymphocytosis | Blood and lymphatic system disorders | 47.11 | 9 | 5.55 | 2.15 |
| Bacteraemia | Infections and infestations | 8.77 | 9 | 3.13 | 1.39 |
| Paralysis | Nervous system disorders | 6.78 | 9 | 2.76 | 1.19 |
| Ageusia | Nervous system disorders | 3.95 | 9 | 1.98 | 0.69 |
| Cognitive disorder | Nervous system disorders | 2.16 | 9 | 1.11 | 0.04 |
| Respiratory syncytial virus infection | Infections and infestations | 10.98 | 8 | 3.46 | 1.42 |
| Acute respiratory distress syndrome | Respiratory, thoracic and mediastinal disorders | 5.04 | 8 | 2.33 | 0.83 |
| Haematological infection | Infections and infestations | 44.5 | 7 | 5.47 | 1.76 |
| Rhinovirus infection | Infections and infestations | 23.43 | 7 | 4.55 | 1.6 |
| Cytomegalovirus viraemia | Infections and infestations | 19.35 | 7 | 4.27 | 1.53 |
| Large intestine perforation | Gastrointestinal disorders | 11.17 | 7 | 3.48 | 1.28 |
| Immunosuppression | Immune system disorders | 9.55 | 7 | 3.25 | 1.18 |
| Covid-19 pneumonia | Infections and infestations | 6.56 | 7 | 2.71 | 0.93 |
| Haemorrhage intracranial | Nervous system disorders | 5.13 | 7 | 2.36 | 0.74 |
| Hypersomnia | Nervous system disorders | 2.72 | 7 | 1.44 | 0.14 |
| Aphasia | Nervous system disorders | 2.51 | 7 | 1.33 | 0.06 |
| Flat affect | Psychiatric disorders | 31.44 | 6 | 4.97 | 1.46 |
| Adenovirus infection | Infections and infestations | 24.93 | 6 | 4.64 | 1.4 |
| Facial paresis | Nervous system disorders | 18.69 | 6 | 4.22 | 1.31 |
| Failure to thrive | Metabolism and nutrition disorders | 12.24 | 6 | 3.61 | 1.14 |
| Hypertransaminasaemia | Hepatobiliary disorders | 11.19 | 6 | 3.48 | 1.1 |
| Immunodeficiency | Immune system disorders | 4.83 | 6 | 2.27 | 0.55 |
| Upper limb fracture | Injury, poisoning and procedural complications | 3.29 | 6 | 1.72 | 0.22 |
| Eating disorder | Psychiatric disorders | 3.13 | 6 | 1.65 | 0.17 |
| Iiird nerve paralysis | Nervous system disorders | 74.75 | 5 | 6.21 | 1.31 |
| Pneumonia pseudomonal | Infections and infestations | 27.67 | 5 | 4.79 | 1.16 |
| Platelet disorder | Blood and lymphatic system disorders | 22.46 | 5 | 4.49 | 1.11 |
| Duodenitis | Gastrointestinal disorders | 17.86 | 5 | 4.16 | 1.05 |
| Cytomegalovirus infection reactivation | Infections and infestations | 15.21 | 5 | 3.92 | 0.99 |
| Staphylococcal bacteraemia | Infections and infestations | 13.47 | 5 | 3.75 | 0.95 |
| Enterocolitis | Gastrointestinal disorders | 10.57 | 5 | 3.4 | 0.85 |
| Intestinal ischaemia | Gastrointestinal disorders | 10.18 | 5 | 3.35 | 0.83 |
| Peripheral sensory neuropathy | Nervous system disorders | 9.77 | 5 | 3.29 | 0.81 |
| Decreased immune responsiveness | Immune system disorders | 5.95 | 5 | 2.57 | 0.53 |
| Malnutrition | Metabolism and nutrition disorders | 5.12 | 5 | 2.36 | 0.42 |
| Tachypnoea | Respiratory, thoracic and mediastinal disorders | 4.21 | 5 | 2.07 | 0.28 |
| Phrenic nerve paralysis | Nervous system disorders | 216.83 | 4 | 7.73 | 0.99 |
| Norovirus infection | Infections and infestations | 41.52 | 4 | 5.37 | 0.89 |
| Reduced facial expression | Nervous system disorders | 33.14 | 4 | 5.05 | 0.86 |
| Parainfluenzae virus infection | Infections and infestations | 28.59 | 4 | 4.83 | 0.84 |
| Pseudomonal sepsis | Infections and infestations | 26.22 | 4 | 4.71 | 0.82 |
| Gastrointestinal toxicity | Gastrointestinal disorders | 10.48 | 4 | 3.39 | 0.56 |
| Appetite disorder | Metabolism and nutrition disorders | 7.1 | 4 | 2.83 | 0.38 |
| Dysgraphia | Nervous system disorders | 6.64 | 4 | 2.73 | 0.35 |
| Escherichia infection | Infections and infestations | 5.69 | 4 | 2.51 | 0.26 |
| Subarachnoid haemorrhage | Nervous system disorders | 4.28 | 4 | 2.1 | 0.08 |
| Electrolyte imbalance | Metabolism and nutrition disorders | 4.03 | 4 | 2.01 | 0.04 |
| Intestinal perforation | Gastrointestinal disorders | 3.96 | 4 | 1.98 | 0.02 |
| Metapneumovirus pneumonia | Infections and infestations | 556.17 | 3 | 9.04 | 0.5 |
| Autoimmune enteropathy | Gastrointestinal disorders | 487.87 | 3 | 8.86 | 0.5 |
| Iiird nerve disorder | Nervous system disorders | 237.68 | 3 | 7.86 | 0.51 |
| Neuralgic amyotrophy | Nervous system disorders | 162.62 | 3 | 7.32 | 0.51 |
| Pulmonary mucormycosis | Infections and infestations | 71.3 | 3 | 6.15 | 0.49 |
| Gastroenteritis escherichia coli | Infections and infestations | 68.16 | 3 | 6.08 | 0.49 |
| Cytokine storm | Immune system disorders | 50.75 | 3 | 5.66 | 0.47 |
| Metapneumovirus infection | Infections and infestations | 46.74 | 3 | 5.54 | 0.46 |
| Cogwheel rigidity | Nervous system disorders | 31.96 | 3 | 4.99 | 0.42 |
| Vith nerve paralysis | Nervous system disorders | 29.62 | 3 | 4.88 | 0.41 |
| Chronic inflammatory demyelinating polyradiculoneuropathy | Nervous system disorders | 27 | 3 | 4.75 | 0.4 |
| Peripheral motor neuropathy | Nervous system disorders | 24.04 | 3 | 4.58 | 0.38 |
| Pneumonia cytomegaloviral | Infections and infestations | 17.69 | 3 | 4.14 | 0.33 |
| Systemic candida | Infections and infestations | 14.38 | 3 | 3.84 | 0.28 |

**Table S7** Search terms used for CAR-T product identification in FAERS

| **Drugs** | **Generic, brand, abbreviated, and development names** |
| --- | --- |
| Breyanzi | lisocabtagene maraleucel，liso-cel，Breyanzi，JCAR017 |
| Kymriah | CTL019，Kymriah，Tisagenlecleucel，tisa-cel |
| Tecartus | brexucabtagene autoleucel，Tecartus，Brexu-Cel，KTE-X19，KTEX19 |
| Yescarta | Yescarta，Axicabtagene Ciloleucel，axi-cel，KTE-C19，KTE C19 |
| Abecma | idecabtagene vicleucel，Abecma，ide-cel，bb2121，BB-2121 |
| Carvykti | Carvykti，ciltacabtagene autoleucel，cilta-cel，JNJ-68284528，JNJ68284528，JNJ-4528，LCAR-B38M |

**Table S8** Category-wise distribution and representative examples of excluded PTs

| **Exclusion category** | **Breyanzi** | **Kymriah** | **Tecartus** | **Yescarta** | **Abecma** | **Carvykti** | **Total, n** | **Representative examples** |
| --- | --- | --- | --- | --- | --- | --- | --- | --- |
| Indication-, disease-background-, neoplasm-, or disease-progression-related terms | 5 | 33 | 13 | 23 | 4 | 16 | 94 | Lymphoma; Disease progression |
| Laboratory or investigation terms | 7 | 58 | 10 | 18 | 13 | 12 | 118 | Platelet count decreased; Serum ferritin increased |
| Surgical/medical procedure- or treatment-process-related terms | 2 | 1 | 2 | 6 | 1 | 7 | 19 | Lymphocyte adoptive therapy; Dialysis |
| Lack-of-efficacy or treatment-response outcome terms | 0 | 3 | 0 | 1 | 0 | 0 | 4 | Treatment failure; Therapy non-responder |
| CAR-T persistence, pharmacodynamic durability, or recovery markers | 0 | 3 | 1 | 1 | 0 | 0 | 5 | B-cell aplasia; Loss of CAR T-cell persistence |
| Product-quality-related terms | 0 | 1 | 0 | 1 | 0 | 4 | 6 | Manufacturing issue; Product packaging issue |
| Other excluded or non-interpretable terms | 0 | 0 | 0 | 0 | 0 | 2 | 2 | Adverse event; Adverse drug reaction |
| **Total** | **14** | **99** | **26** | **50** | **18** | **41** | **248** | **—** |

**Figure S1** Signal detection results and forest plots of RORs (95% CI) for the major AE signals associated with Breyanzi

**Figure S2** Signal detection results and forest plots of RORs (95% CI) for the major AE signals associated with Kymriah

**Figure S3** Signal detection results and forest plots of RORs (95% CI) for the major AE signals associated with Tecartus

**Figure S4** Signal detection results and forest plots of RORs (95% CI) for the major AE signals associated with Yescarta

**Figure S5** Signal detection results and forest plots of RORs (95% CI) for the major AE signals associated with Abecma

**Figure S6** Age-subgroup analysis of positive signals for six CAR-T products

**Figure S7** Sex-subgroup analysis of positive signals for six CAR-T products

**Figure S8** Outcome-subgroup analysis of positive signals for six CAR-T products
